# Chemical response mechanisms of *Solanum rostratum* under different selection pressures of herbivores

**DOI:** 10.3389/fpls.2026.1955283

**Published:** 2026-09-30

**Authors:** Linlin Yuan, Xiaomin Yang, Zhiqun Sui, Yangyang Zhang, Zhixiang Liu, Zhifu Guo, Yulong Feng

**Affiliations:** College of Bioscience and Biotechnology, Shenyang Agricultural University, Shenyang, China

**Keywords:** generalist herbivores, *Helicoverpa armigera*, invasive plants, *Leptinotarsa decemlineata*, qualitative defense, quantitative defense, *Solanum rostratum*, specialist herbivores

## Abstract

The revised Evolution of Increased Competitive Ability (EICA) hypothesis suggests that when invasive plants escape the selective pressure exerted by specialist herbivores, they reallocate their resources: reducing investment in defense against specialists while increasing defense against generalist herbivores. Such shifts in defense strategies are mediated by chemical defenses underpinned by plant secondary metabolites. However, how quantitative and qualitative defensive metabolites evolve in response to this selective shift remains poorly understood. Focusing on plant secondary metabolites, *Solanum rostratum* was selected as the focal species. A common garden experiment was conducted to assess plant growth and defense traits, followed by bioassays using its specialist herbivore *Leptinotarsa decemlineata* and generalist herbivore *Helicoverpa armigera* to evaluate defensive activity. Quantitative and qualitative defensive metabolites from *S. rostratum* were isolated and identified, and their defensive functions were validated. Differences in these metabolites across populations and their interrelationships were compared, and their correlations with plant growth were analyzed. Under herbivory pressure from generalist herbivores, *S. rostratum* shifted resource allocation away from quantitative defensive metabolites toward both qualitative defensive metabolites and vegetative growth. In contrast, under combined herbivory pressure from both specialist and generalist herbivores, *S. rostratum* maintained high levels of quantitative defensive metabolites, and its growth showed no significant evolutionary divergence. Distinct evolutionary patterns of quantitative and qualitative defensive metabolites were exhibited by invasive plants, and a resource trade-off between growth and defense was demonstrated. These findings advance understanding of the invasion mechanisms of alien plant species.

## 1 Introduction

In recent years, with the acceleration of economic globalization and trade liberalization, the invasion of alien plants has become a far-reaching global problem. The resulting problems in agriculture, forestry, ecological environment, social economy, and human health are becoming increasingly serious (Bonnamour et al., 2021). Plants and insects are two important components of terrestrial ecosystems. Over a long period of co-evolution, the two have developed many complex adaptive mechanisms. Therefore, it is of great significance to study the interactions between alien plants and herbivorous insects to reveal the invasion mechanisms of alien plants (Kariñho-Betancourt, 2020; Liang et al., 2025).

In order to reveal the invasion mechanisms of alien plants, a variety of hypotheses have been put forward. The Enemy Release Hypothesis (ERH) holds that when alien plants arrive in new habitats, they escape the control of herbivorous insects with which they co-evolved in their native ranges and form competitive release in introduced ranges, resulting in range expansion and population increase of alien plants (Kennedy et al., 2002; Brian and Catford, 2023). In addition, there is a trade-off in the resource allocation of plants between defense and growth (Monson et al., 2021). In view of this trade-off relationship, some scholars have proposed the Evolution of Increased Competitive Ability (EICA) hypothesis. It suggests that alien plants in the new habitat can produce an evolutionary response to the lack of specialist herbivores, reduce the defense abilities against specialist herbivores, and transfer the resources that originally maintained defense to growth and reproduction, thus improving their competitiveness (Blossey and Nötzold, 1995). Although numerous studies have verified the EICA hypothesis, there are also many inconsistent or even contrary reports (Felker-Quinn et al., 2013; Egbon et al., 2020). In fact, alien plants only escape from specialist herbivores in introduced ranges, and are still affected by generalist herbivores (Parker et al., 2006). However, Müller-Schärer et al. (2004) proposed a revised EICA hypothesis based on the changes in the composition of enemies after the invasion of alien plants and the different defense mechanisms of alien plants against specialist and generalist herbivores. It suggests that alien plants may only reduce the defense against specialist herbivores, but not necessarily reduce the defense against generalist herbivores, and the change of defense strategy is mediated by chemical effects of secondary metabolites.

Chemical defense of plants can be divided into quantitative defense (high-cost) and qualitative defense (low-cost). Quantitative defensive metabolites refer to a class of compounds in plants that can affect the digestion and utilization of food by herbivores, resulting in slow growth and reduced fertility of insects. These defensive metabolites are mainly phenolic compounds, including lignin, tannin and simple phenolic acids. Qualitative defensive metabolites refer to a class of compounds in plants that can cause toxicity, avoidance, and antifeedant by herbivores. These defensive metabolites are mainly alkaloids (Müller-Schärer et al., 2004; Cao et al., 2009). In the native ranges, the contents of qualitative defensive metabolites of alien plants may be at an intermediate or low level under the common pressures of specialist and generalist herbivores. On the other hand, in the introduced ranges, the contents of quantitative defensive metabolites of alien plants may decrease due to the lack of selective pressure of specialist herbivores, and the resources will be compensated by increasing the contents of qualitative defensive metabolites and enhancing the growth and reproduction abilities of plants (Müller-Schärer et al., 2004; Joshi and Vrieling, 2005).

*Solanum rostratum* Dunal. (Solanaceae) is a malignant invasive weed, native to North America and now spread to many countries and regions including Europe, Asia, Africa and Oceania (Zhong et al., 2009). The species reproduces rapidly, exhibits strong adaptability and spreads quickly. It competes with native plants for sunlight, nutrients, water, and living space, causing severe harm to crops and severely damaging the ecological environment and biodiversity of introduced ranges (Du et al., 2024; Xiang et al., 2025). *S. rostratum* can also produce solanine, a neurotoxin that has a paralyzing effect on the central nervous system, endangering the health of humans and livestock. It has become a harmful alien weed that urgently needs to be controlled and eradicated (Rushing et al., 1985; Wei et al., 2010). Furthermore, studies have shown that *S. rostratum* is rich in alkaloid and phenolic acid defensive metabolites (Wang et al., 2015; Liu et al., 2020, Liu et al., 2021). Therefore, this plant is suitable for research on quantitative and qualitative defensive metabolites.

*Leptinotarsa decemlineata* (Coleoptera: Chrysomelidae) is a world-famous and devastating invasive pest (Gao et al., 2022). It mainly attacks potatoes and other Solanaceae crops. Both *L. decemlineata* and *S. rostratum* are native to North America and have now spread to more than forty countries (Zhang et al., 2013; Shi et al., 2023). The host range of *L. decemlineata* is relatively narrow, and its original host is *S. rostratum*. Moreover, the feeding area and the preference for egg-laying on *S. rostratum* are both significantly larger than those of crops of Solanaceae family such as *S. tuberosum*, *S. melongena* and *S. nigrum* (Izzo et al., 2014). Meanwhile, studies have also shown that *L. decemlineata* has a strong ability to recognize its original host-*S. rostratum*. This recognition ability is not lost even if it has been separated from its original host for a long time. It is a specialist herbivore of *S. rostratum* (Horton et al., 1988; Izzo et al., 2014).

*Helicoverpa armigera* (Lepidoptera: Noctuidae) is one of the most widely distributed and harmful polyphagous agricultural pests worldwide. It is widely distributed in regions such as Asia, Europe, Africa, and Oceania (Afrazeh and Sendi, 2025). It occurs in almost all major crop-producing areas of China and poses a threat to more than two hundred types of crops, including cotton, corn, and tomatoes (Jin et al., 2023). It mainly feeds on the tender leaves and flower buds of plants. When major crops (such as *S. tuberosum* and *S. lycopersicum*) are scarce, *S. rostratum* can serve as a transitional food source for them. Especially in the barren lands and farmland edges where *S. rostratum* is abundantly distributed, *H*. *armigera* could actively choose it as a host for laying eggs and feeding their larvae. It is a generalist herbivore of *S. rostratum* (Tay et al., 2013; Liu et al., 2020; Riaz et al., 2021).

However, under different selection pressures of herbivores, has the defense strategy of *S. rostratum* undergone any changes? Which defensive metabolites constitute the material basis for the qualitative and quantitative defense mechanisms of *S. rostratum*? How do these small molecules function in the trade-off relationship between the growth and the defense of *S. rostratum*? These issues remain unknown as of now. Only after a clear understanding of these scientific issues can we truly understand the chemical response and evolutionary mechanisms of invasive plants under different selection pressures of herbivores.

## 2 Materials and methods

### 2.1 Field surveys and sampling

In September 2022, field surveys were conducted at twelve sites in Liaoning Province and Xinjiang Uygur Autonomous Region of China (**Figure 1**; **Supplementary Table 1**). Based on field observations, it was found that the growth of different populations of *S. rostratum* in Liaoning were significantly better than those in Xinjiang (**Supplementary Figure 1**). In addition, both the specialist and generalist herbivores (*L. decemlineata* and *H. armigera*) of *S. rostratum* are distributed in different habitats of Xinjiang. However, only *S. rostratum* and its generalist herbivores (*H. armigera*) are widely distributed in Liaoning, and *L. decemlineata* has not been found (**Supplementary Figure 2**). Thus, *S. rostratum* from Xinjiang and Liaoning provided an excellent system for studying the pattern of insect selection pressure in this research.

**Figure 1:**
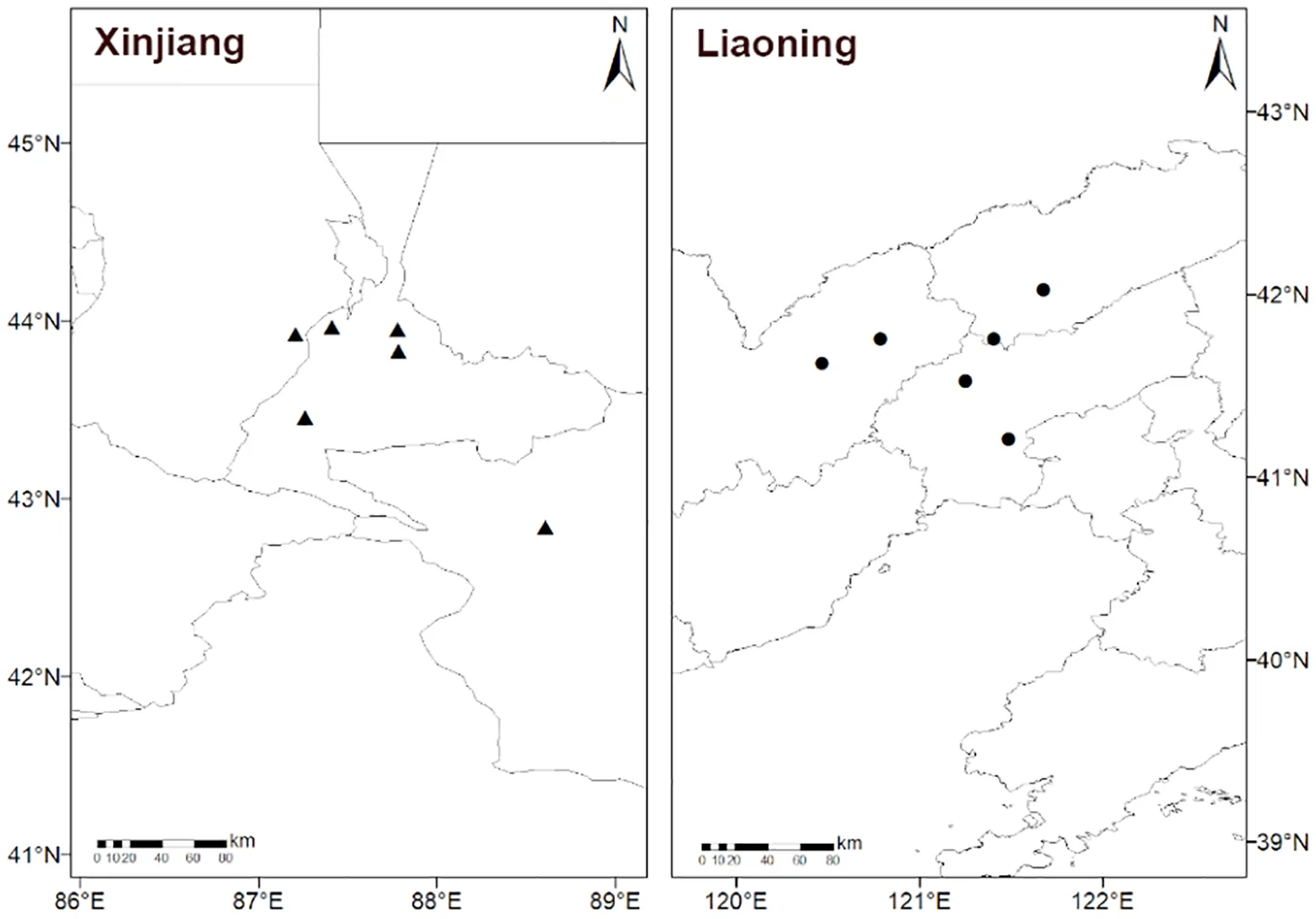
Geographical distributions map of different populations of *S. rostratum* in Xinjiang and Liaoning.

Seeds of *S. rostratum* were collected from six populations in Liaoning and six populations in Xinjiang, China. In each population, twenty individual plants were randomly selected, and 5–8 mature fruits (approximately 50–80 full seeds) were harvested per individual to ensure sufficient seed yield and representative sampling for subsequent experiments. All seeds were then stored separately at 4 °C. For chemical analyses, above-ground parts of *S. rostratum* were collected from Fuxin, Liaoning Province, China (121°26′ E, 41°45′ N) in September 2022.

### 2.2 Extraction and isolation

The above-ground parts of dried *S. rostratum* plants (40 kg) were crushed and immersed in 75% ethanol. Extraction was performed twice by the heat reflux method (2 h each time). The concentrated ethanol extract was successively partitioned with petroleum ether and ethyl acetate. The ethyl acetate fraction was then passed through silica chromatography eluted with a gradient of CH_2_Cl_2_:CH_3_OH, and subsequently divided into seven fractions (Fr. A–G). By comprehensively utilizing multiple column chromatography techniques (including silica gel column chromatography, gel column chromatography, macroporous resin chromatography and semi-preparative RP-HPLC), thirty-one compounds were successfully separated (**Supplementary Figure 3**). Their structures were identified by ^1^H and ^13^C NMR (Bruker, Karlsruhe, Germany).

### 2.3 The common garden experiment

In early May 2024, a common garden experiment was conducted at Shenyang Agriculture University (123°33′E, 41°48′N) where the mean elevation is 59 m, mean annual temperature is 8.1 °C, and mean annual precipitation is 722 mm. Seeds for this experiment were collected from six locations in Liaoning Province and six locations in Xinjiang Uygur Autonomous Region of China. Fifty seeds were randomly selected from each population for germination. For seed dormancy breaking, seeds were first soaked in 0.6 mmol L^-1^ gibberellin (GA_3_) solution in the dark at 25 °C for 24 h, then rinsed with distilled water 3–5 times to remove residual gibberellin, and finally surface-dried with filter paper prior to sowing. This protocol was optimized for *S. rostratum* by Wei et al (Wei et al., 2010). and resulted in a germination rate of approximately 85% across all populations. The substrate made of soil and sand (6:4, *v*/*v*) was placed into 32-cell plug trays for seed emergence. One week after seedling emergence, twelve healthy and uniformly sized seedlings (each originating from a distinct maternal individual) were randomly selected per population and transplanted into individual 10 L pots under identical environmental conditions. Of these, six were retained for subsequent experiments, and the other six were used for initial biomass measurement. The sample size of six seedlings per population was determined by referring to previous relevant common garden studies (Zhao et al., 2020; Tian et al., 2021; Yin et al., 2023; Gallego-Tévar et al., 2024). The transplanted seedlings were grown in the same peat soil/sand mixture with 5 g of slow-release fertilizer (N:P:K = 1:1:1) added to each pot. All pots were watered every day.

In early August 2024, the 3rd to 5th fully expanded, mature and healthy functional leaves from the top of each individual plant were uniformly selected for physiological measurements. The maximum light-saturated photosynthetic rate (P_max_) was determined using a portable photosynthesis system (Li-Cor Environmental, Lincoln, USA). The light intensity in the leaf chamber was set to 1500 *μ*mol m^-2^ s^-1^, the CO_2_ concentration of the reference chamber was set to 380 *μ*mol mol^-1^, and the leaf temperature was set to 27 °C. All leaves were fully light-adapted under saturated light before measurement. Leaf relative chlorophyll content was measured with a SPAD chlorophyll meter (Konica Minolta, Tokyo, Japan).

In mid-August 2024, plant height, crown width, and basal stem diameter were measured before plant harvesting. Plant height was defined as the vertical distance from the pot soil surface to the highest growing apex of each individual. Basal stem diameter was measured at the stem base immediately adjacent to the pot soil surface. Crown width was measured under natural growth status by determining the maximum canopy extensions along two perpendicular east-west and north-south directions with a soft tape, and the average value was taken as the final crown width per plant. The photosynthetic leaf area and the total single-sided leaf area were measured using an Area Meter (Li-Cor Environmental, Lincoln, USA). Subsequently, the leaves were dried at 60 °C for 72 h to constant weight and then weighed. The sediment on the roots was washed off with clean water. Then, the aboveground and underground parts of the plants were separated, put into marked envelopes respectively, and dried in an oven at 60 °C for 72 h. The aboveground and root biomass of the plants were weighed, and the total biomass was calculated. The average relative growth rate (RGR) was calculated using Poorter’s method (Poorter, 1999): $\text{RGR}=\frac{{(\text{lnW}}_{2}–\text{ln}\bar{{\text{W}}_{1}})}{\Delta t}$. Here, $\bar{{\text{W}}_{1}}$ represents the arithmetic mean of the initial biomass (g) of the plants, W_2_ represents the total biomass (g) of the plants at harvest, and Δt represents the time interval (days) between the two measurements.

### 2.4 Insect bioassays

In order to explore the chemical defense abilities of compounds isolated from *S. rostratum*, antifeedant and growth inhibitory activity assays were performed against *H. armigera* and *L. decemlineata*. Under laboratory conditions, the larvae of *H. armigera* (Keyun, Qiyuan, China) were reared on artificial diets. The rearing environment was maintained at a temperature of 27 ± 2 °C, a relative humidity of 45 ± 5%, and a photoperiod consisting of 16 h of light followed by 8 h of darkness. The antifeedant activity was determined using the non-choice bioassay method (Liu et al., 2020). For bioassay validity, leaf disks were prepared from the respective preferred host plant of each insect species: *Brassica chinensis* for *H. armigera*, and *S. tuberosum* for *L. decemlineata*, consistent with their host specificity and feeding habits. Fresh leaf disks were punched out from the leaves of *B. chinensis* using a borer with a diameter of 1.0 cm. Treated leaf disks were coated with 20 *μ*L of methanol solution containing the test compounds (**1**–**31**) (100 *μ*g mL^-1^) or ethanol extracts (1 mg mL^-1^). The control leaf disks were treated with an equal volume of methanol. After air drying, four test leaves were arranged alternately in a Petri dish with a diameter of 9 cm, and a moistened filter paper was placed at the bottom of each dish. Two third-instar larvae of *H. armigera* of similar size were placed in the center of each Petri dish after starvation for 6 h. Each treatment was established with three replicates. When approximately 90% of the blank control leaf discs had been consumed, the larvae were carefully removed from the Petri dish, and the cumulative leaf area consumption was accurately measured. The antifeedant activity of *L. decemlineata* (the third-instar larvae of *L. decemlineata* provided by Associate Professor Li Chao from Xinjiang Agricultural University) was determined using the same method. The leaf discs were replaced with *S. tuberosum* leaves. The antifeedant rate (AR) was calculated using the formula: $\text{AR}(\%)=\frac{\left({\text{A}}_{\text{C}}–{\text{A}}_{\text{T}}\right)}{{\text{A}}_{\text{C}}}\times 100$, where A_C_ and A_T_ are the consumed areas of the blank control and leaf discs treated with ethanol extracts or compounds, respectively.

The growth inhibitory assay of *L. decemlineata* was determined using the method described above with some modifications. Ten treated leaf discs and one third-instar larva of *L. decemlineata* (starved for 6 h) were placed in each Petri dish. The growth inhibitory assay of *H. armigera* was carried out using the diet incorporation method (Kumar et al., 2023). The artificial diet (Keyun, Qiyuan, China) was uniformly mixed with ethanol extracts (1 mg mL^-1^) or compounds (100 *μ*g mL^-1^). And the control groups were treated with an equal volume of methanol. Third instar larvae of *H. armigera* (starved for 6 h) with uniform weight, size, and stage were placed in each Petri dish and fed under the controlled conditions of 27 ± 2 °C in temperature, 45 ± 5% in relative humidity, and a photoperiod consisting of 16 h of light followed by 8 h of darkness. The mixed feed containing the ethanol extracts or compounds were replaced every other day. Each experiment was repeated at least three times. The *L. decemlineata* and *H. armigera* were both accurately weighed at 0 h and 72 h, respectively. The growth inhibitory rate (GR) was calculated using the formula: $\text{GR}(\%)=\frac{\left({\text{W}}_{\text{C}}–{\text{W}}_{\text{T}}\right)}{{\text{W}}_{\text{C}}}\times 100$, where W_C_ and W_T_ are the weight gains after feeding the blank control diet and the diet treated with the ethanol extracts or compounds, respectively.

### 2.5 Chemical analyses of different populations

The above-ground parts of dried *S. rostratum* from all populations were crushed and extracted with 75 % ethanol (sample to solvent ratio of 1:2). Ultrasonic extraction was performed for 2 h for each time, and the extraction procedure was repeated for three times. The filtrate was collected after extraction and concentrated to obtain ethanol extracts for subsequent UPLC-MS/MS analysis. The defensive metabolites in different populations were qualitatively analysed by comparison with retention time and mass spectrum of compounds 1–31 using a UPLC-MS/MS (Agilent, Santa Clara, USA) equipped with an EC-C18 column (50 mm × 2.1 mm, 1.9 *μ*m, 0.5 mL min^-1^, 40°C, Agilent, Santa Clara, USA). In addition, quantitative analysis of defensive metabolites was carried out using the external standard calibration method. Serial concentrations of compounds 1–31 were prepared to establish standard curves, and the contents of defensive metabolites in samples were quantified according to peak area comparison (**Supplementary Table 2**).

### 2.6 Statistical analyses

All data analyses were performed in R 4.1.0 (R Development Core Team 2021). Before conducting the data analysis, tests for normal distributions and homogeneity of variances were performed on all datasets. In the common garden experiment, linear mixed models (LMMs) were constructed to evaluate the effect of site (Liaoning and Xinjiang) on growth traits of *S. rostratum*, with site set as a fixed effect and population nested within site as a random effect. The random effect of population was included to account for intraspecific variation and improve the precision of site-level comparisons. Models were fitted using the lmer function in the lme4 package, and the lmerTest package was used to test the significance of model parameters. This analysis included twelve total populations (six from Liaoning and six from Xinjiang), with six biological replicates per population, totaling seventy-two experimental plants (thirty-six from Liaoning and thirty-six from Xinjiang). To compare the differences in the contents (qualitative and quantitative) of defensive metabolites and biological activities (antifeedant and growth inhibitory activities against *H. armigera* and *L. decemlineata*) of *S. rostratum* from different locations (Liaoning and Xinjiang), generalized linear models (GLMs) with a Gaussian distribution were conducted; models were constructed using the glm function from the stats package in R. In the model setting, site (Liaoning and Xinjiang) was designated as the independent variable, and contents of defensive metabolites or biological activities were used as the dependent variable. Among them, the analysis of defensive metabolite content matched the population and replicate design of the common garden experiment (a total of thirty-six samples: six populations in Liaoning × three biological replicates, six populations in Xinjiang × three biological replicates), while each treatment in the biological activity analysis included three biological replicates (independent preparations of extracts/compounds). Besides, Pearson correlation analyses were used to examine the correlations between the contents of defensive metabolites and plant growth traits, as well as between the biological activities of different populations and plant growth traits.

## 3 Results

### 3.1 Chemical structure identification

Secondary metabolites, including alkaloids and phenolic acids constitute major chemical defensive metabolites within Solanaceae plants (Stamp and Yang, 1996; Cao et al., 2009; Sun et al., 2015). In the present study, secondary metabolites of *S. rostratum* were isolated and identified to yield candidate defensive metabolites. All the thirty-one compounds (1–31) were identified as 3-(4-hydroxy-3-methoxyphenyl)-*N*-[2-(4-hydroxyphenyl)-2-methoxyethyl]-acrylamide (1) (Lee et al., 2001), *N*-[2-hydroxy-2-(4-hydroxyphenyl)-ethyl]-ferulamide (2) (DellaGreca et al., 2006), *N*-*trans*-feruloyltyramine (3) (Sun et al., 2015), *p*-methoxycinnamoylamide (4) (Nishioka et al., 1997), phenylethylamine A (5) (Liu et al., 2020), 3-(4-hydroxyphenyl)-*N*-[2-(4-hydroxyphenyl)-2-methoxyethyl]-acrylamide (6) (Liu et al., 2020), *N*-*trans*-*p*-coumaroylnoradrenline (7) (Sun et al., 2015), *N*-*trans*-*p*-coumaroyloctopamine (8) (Liu et al., 2020), *N*-*trans*-*p*-coumaroyl-3′, 4′-dihydroxyphenylethylamine (9) (Liu et al., 2020), *trans*-*N*-coumaroyltyramine (10) (Al-Taweel et al., 2012), *N*-*cis*-feruloyltyramine (11) (King and Calhoun, 2005), *cis*-*N*-phydroxycinnamoyl-7’-methoxyethyltyramine (12) (Wang et al., 2017), *cis*-*N*-*p*-coumaroylnoradrenaline (13) (López-Gresa et al., 2011), *N*-*cis*-*p*-coumaroyloctopamine (14) (Liu et al., 2020), *cis*-*N*-coumaroyltyramine (15) (Kim et al., 2005), phenylpropylcyclamide A (16) (Liu et al., 2023), adenine ribonucleoside (17) (Dang et al., 2010), 2’-deoxythymidine (18) (Jones et al., 1970), vanillic acid (19) (Huang et al., 2022), gallic acid (20) (Nguyen et al., 2013), protocatechuic acid (21) (Kuo et al., 2019), syringic acid (22) (Chen et al., 2010), 3, 5-dihydroxybenzoic acid (23) (Kalinowska et al., 2021), *p*-hydroxybenzoic acid (24) (Chen et al., 2010), 3-hydroxybenzoic acid (25) (He et al., 2011), salicylic acid (26) (Visca et al., 1993), ferulic acid (27) (Woranuch and Yoksan, 2013), caffeic acid (28) (Exarchou et al., 2001), *trans* - 3-hydroxycinnamic acid (29) (Nakazawa and Ohsawa, 1998), *p*-coumaric acid (30) (Kort et al., 1996), 3, 4-dihydroxyphenylacetic acid (31) (Li et al., 2021), respectively (**Figure 2**; **Supplementary Tables 3**–**14**). Based on structural features and published phytochemical records for Solanaceae species, compounds 1–18 were identified as alkaloid type defensive metabolites, representing putative qualitative defensive metabolites (He et al., 2011; Sun et al., 2015; Liu et al., 2020). Additionally, compounds 19–31 were identified as phenolic acid compounds, representing putative quantitative defensive metabolites.

**Figure 2:**
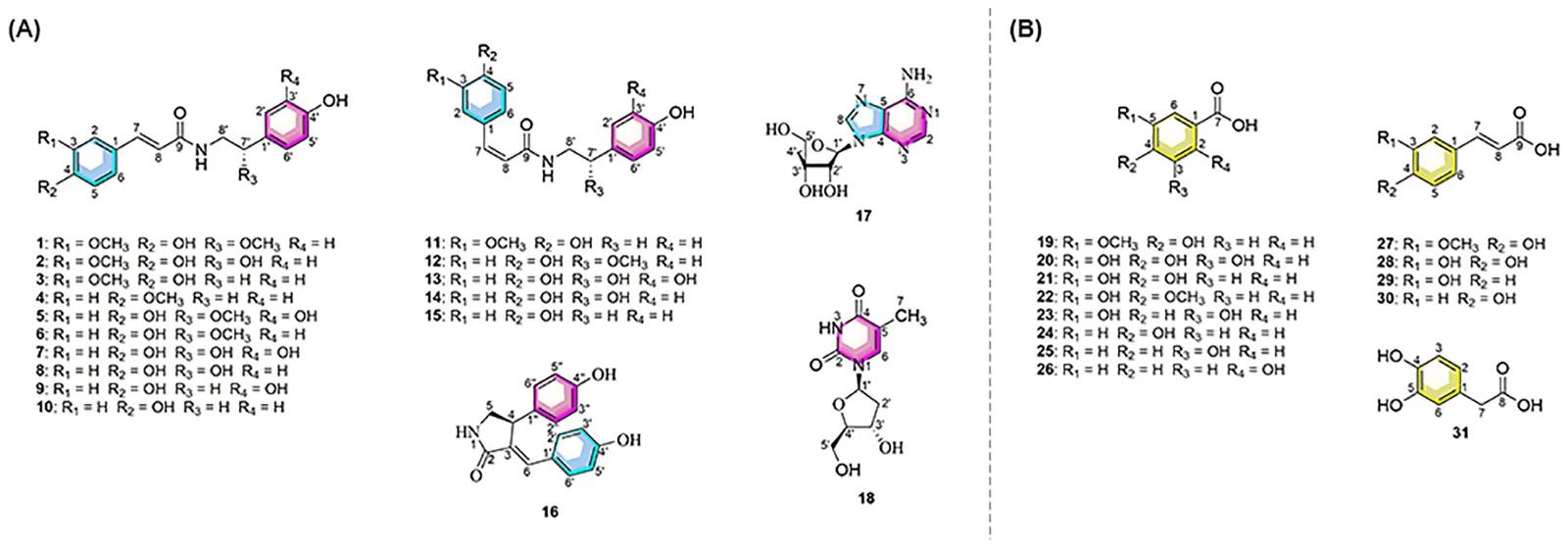
The chemical structures of qualitative and quantitative defensive metabolites. **(A)** The chemical structures of qualitative defensive metabolites (1−18). **(B)** The chemical structures of quantitative defensive metabolites (19−31).

### 3.2 Measurement of plant growth traits

There were significant differences between the Liaoning populations and the Xinjiang populations in all the measured growth traits. Compared with Liaoning populations, Xinjiang populations had lower leaf relative chlorophyll content (*t* = -3.57, *df* = 10, *p* < 0.01), light-saturated photosynthetic rate (*t* = -9.476, *df* = 10, *p* < 0.001), specific leaf area (*t* = -4.928, *df* = 10, *p* < 0.001), aboveground biomass (*t* = -11.46, *df* = 10, *p* < 0.001), root biomass (*t* = -13.10, *df* = 10, *p* < 0.001), crown width (*t* = -22.42, *df* = 10, *p* < 0.001), plant height (*t* = -4.56, *df* = 10, *p* < 0.01), basal diameter of the stem (*t* = -14.10, *df* = 10, *p* < 0.001), total biomass (*t* = -10.72, *df* = 10, *p* < 0.001) and average relative growth rate (*t* = -5.605, *df* = 10, *p* < 0.001), respectively (**Figure 3**; **Supplementary Table 15**). The result indicated that under common garden condition, the Xinjiang populations of *S. rostratum* grew more poorly than the Liaoning populations.

**Figure 3:**
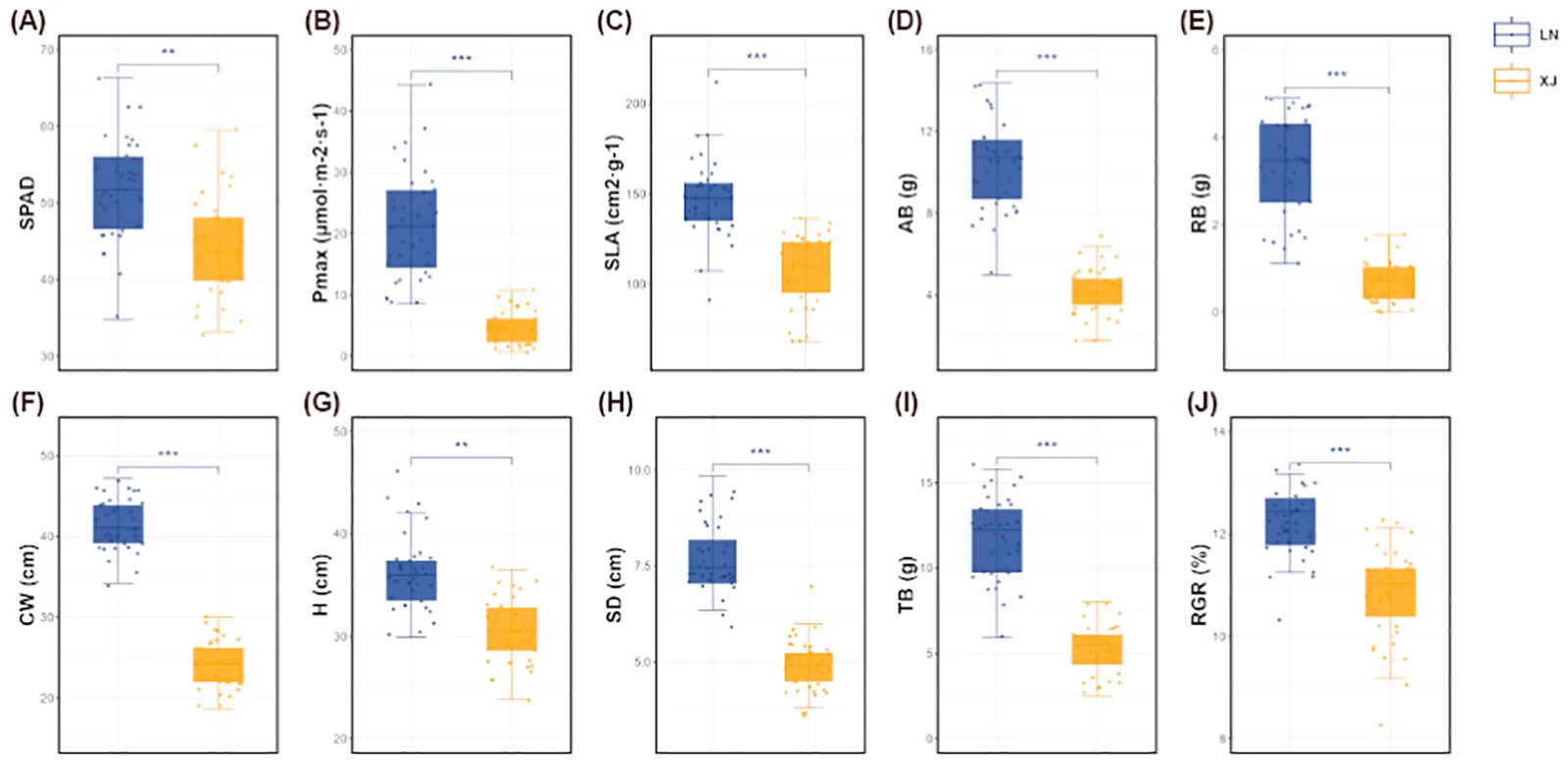
The growth differences of different populations of *S. rostratum* in Liaoning and Xinjiang in the common garden experiment. **(A)** Leaf relative chlorophyll content, SPAD. **(B)** Maximum light-saturated photosynthetic rate, P_max_. **(C)** Specific leaf area, SLA. **(D)** Aboveground biomass, AB. **(E)** Root biomass, RB. **(F)** Crown width, CW. **(G)** Plant height, **(H)** Basal diameter of the stem, SD. **(I)** Total biomass, TB. **(J)** Average relative growth rate, RGR. n = 6 biological replicates per population. Blue and yellow represent the populations of Liaoning and Xinjiang, respectively. *p < 0.05, **p < 0.01 and ***p < 0.001.

### 3.3 Antifeedant and growth inhibitory activities

The ethanol extracts and compounds exhibited different levels of antifeedant and growth inhibitory effects. The antifeedant and growth inhibitory activities of the ethanol extracts from Xinjiang populations of *S. rostratum* against *L. decemlineata* were significantly higher than those of the Liaoning populations, respectively (*t* = 5.78, *df* = 10, *p* < 0.001; *t* = 6.03, *df* = 10, *p* < 0.001). In contrast, the antifeedant and growth inhibitory activities of the ethanol extracts from Xinjiang populations of *S. rostratum* against *H. armigera* were significantly lower than those of the Liaoning populations, respectively (*t* = -8.76, *df* = 10, *p* < 0.001; *t* = -3.30, *df* = 10, *p* < 0.01) (**Figures 4A–C**; **Supplementary Table 16**). The result showed that, compared with the Liaoning populations, the Xinjiang populations of *S. rostratum* had higher defensive ability against specialist herbivores but lower defensive ability against generalist herbivores. The antifeedant and growth inhibitory rates of compounds 1–31 (100 *μ*g mL^-1^) against *H. armigera* ranged from 30.01 ± 6.55 to 79.47 ± 5.31% and 11.08 ± 1.18 to 87.56 ± 3.49%, respectively. Likewise, the antifeedant and growth inhibitory rates of compounds 1–31 against *L. decemlineata* ranged from 6.11 ± 0.79 to 93.20 ± 3.90% and 5.79 ± 2.64 to 75.33 ± 3.72%, respectively (**Figure 4D**).

**Figure 4:**
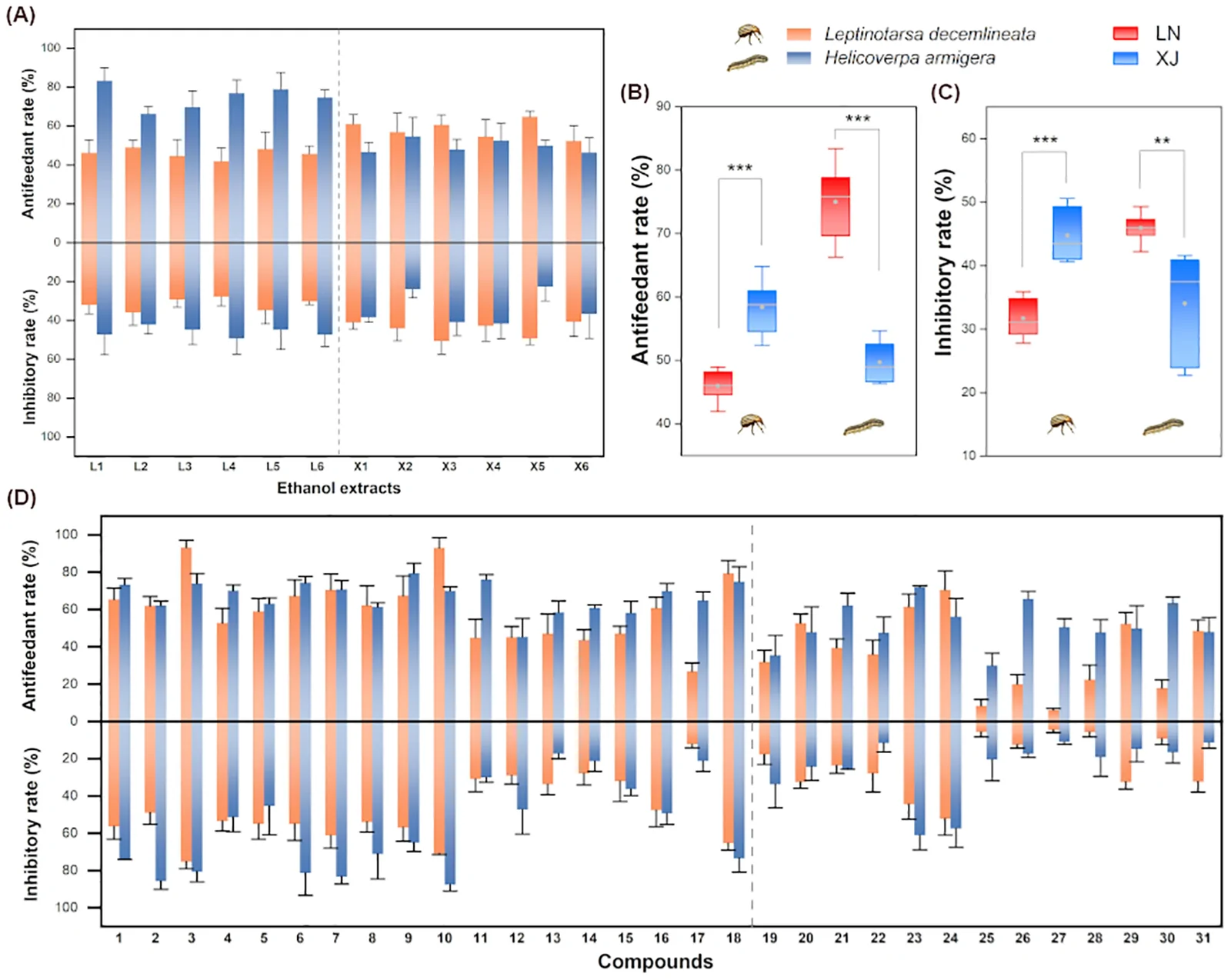
Chemical defense abilities of ethanol extracts and compounds from *S. rostratum* against *L. decemlineata* and *H. armigera*. **(A)** Antifeedant and growth inhibitory activities of ethanol extracts of *S. rostratum* from different populations against *L. decemlineata* and *H. armigera*. **(B)** Significance analyses of ethanol extracts from different populations in Liaoning and Xinjiang on the antifeedant activities of *L. decemlineata* and *H. armigera*, respectively. **(C)** Significance analyses of ethanol extracts from different populations in Liaoning and Xinjiang on the growth inhibitory activities of *L. decemlineata* and *H. armigera*, respectively. **(D)** Antifeedant and growth inhibitory activities of compounds from *S. rostratum* against *L. decemlineata* and *H. armigera*. Data are mean ± SE, n=3. *p < 0.05, **p < 0.01 and ***p < 0.001.

### 3.4 Chemical analyses

Through component identification of ethanol extracts from different populations of *S. rostratum* in Liaoning and Xinjiang, it was found that most of the isolated compounds (2–21, 23, 24, 26–28, 30, 31) could be detected. Content measurement revealed that the contents of qualitative defensive metabolites in different populations of *S. rostratum* in Liaoning were significantly higher than those in Xinjiang, while the contents of quantitative defensive metabolites were significantly lower than those in Xinjiang (**Supplementary Figure 4**; **Supplementary Table 17**).

### 3.5 Correlation analyses between growth and defense

The defense abilities of the ethanol extracts of *S. rostratum* against *L. decemlineata* showed significant negative correlations with the growth traits of *S. rostratum*, while the defense abilities against *H. armigera* showed significant positive correlations (**Figure 5**; **Supplementary Table 18**). Meanwhile, the contents of qualitative defensive metabolites of *S. rostratum* generally showed significant positive correlations with the growth traits of *S. rostratum*, while the contents of quantitative defensive metabolites generally showed significant negative correlations (**Figure 5**; **Supplementary Tables 19**, **20**). The above results indicated that under the common pressures of specialist and generalist herbivores, the quantitative and qualitative defensive metabolites of *S. rostratum* had undergone significant changes, and these changes were closely related to the growth of this plant.

**Figure 5:**
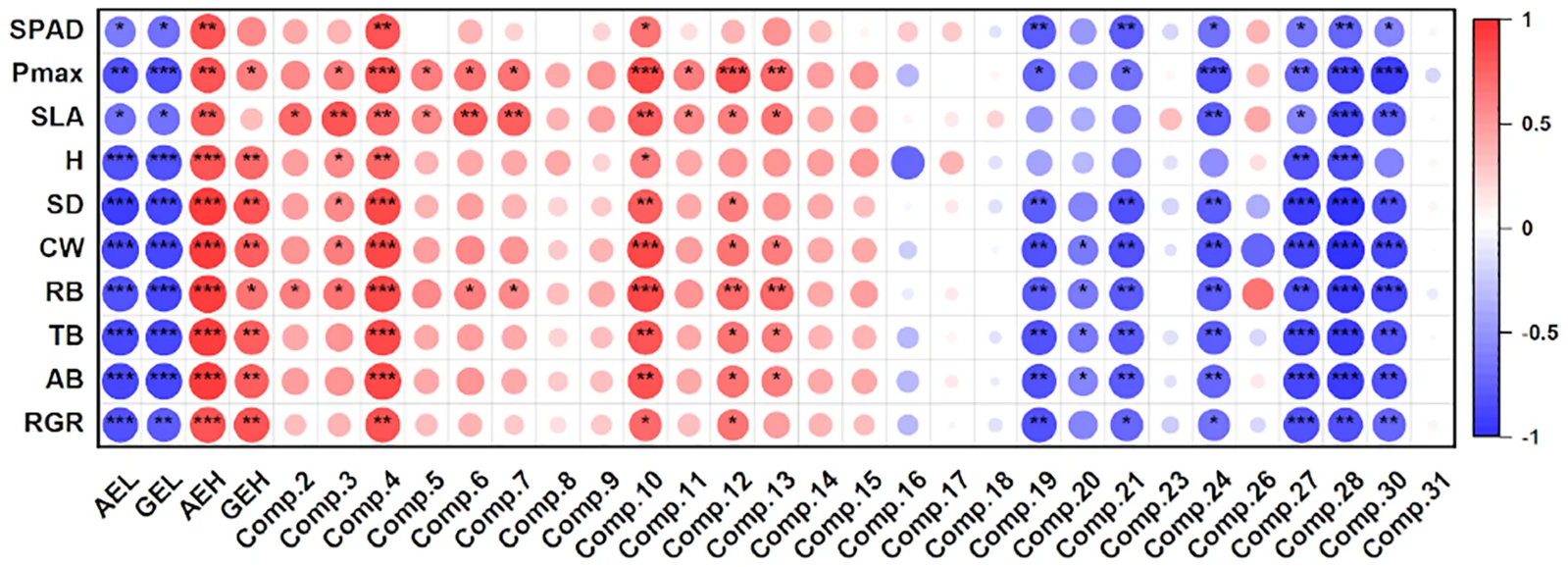
The correlations between the growth traits of different populations of *S. rostratum* and its defense abilities against *L. decemlineata* and *H. armigera*, as well as the contents of different defensive metabolites. In the image, the size of the circles represents the magnitude of the correlation coefficient, and red and blue represent positive correlation and negative correlation, respectively. SPAD, leaf relative chlorophyll content; Pmax, maximum lightsaturated photosynthetic rate; SLA, specific leaf area; H: plant height; SD, basal diameter of the stem; CW, crown width; RB, root biomass; TB, total biomass; AB, aboveground biomass; RGR, average relative growth rate; AEL, antifeedant activities of ethanol extracts against *L. decemlineata*; GEL, growth inhibitory activities of ethanol extracts against *L. decemlineata*; AEH, antifeedant activities of ethanol extracts against *H. armigera*; GEH, growth inhibitory activities of ethanol extracts against *H. armigera*. *p < 0.05, **p < 0.01 and ***p < 0.001.

## 4 Discussion

### 4.1 Selection-driven population growth and defense differentiation

Field surveys confirmed that both the specialist and generalist herbivores of *S. rostratum* occur in Xinjiang. In Liaoning, only the generalist herbivore is widely distributed, and no *L. decemlineata* populations were detected (**Supplementary Figure 2**). Common garden experiments further revealed that under uniform environmental conditions and in the absence of herbivores, the Liaoning and Xinjiang populations still maintained significant differences in growth and defense traits (**Figure 3**). The persistence of trait differences under common garden conditions suggested a partial genetic basis for population divergence, although maternal effects, seed provisioning, and transgenerational epigenetic effects from field collected seeds could not be fully ruled out. Multigeneration common garden experiments would be required for definitive confirmation of evolutionary genetic divergence. Maternal effects were minimized by sampling seeds from 20 maternal plants per population and standardizing germination conditions. Accounting for these limitations, findings were interpreted against well-known ecological hypotheses. From the perspective of the ERH, Liaoning populations had escaped from their long-term co-evolved specialist herbivores and experienced partial enemy release. In addition, in line with the core logic of the revised EICA hypothesis, plants have a finite pool of available resources. When the compositions of herbivores change, plants adjust their defense resource allocation strategies to achieve an optimal trade-off between growth and defense (Zhu et al., 2026).

Furthermore, Liaoning and Xinjiang also have inherent geographical differences in abiotic environmental factors such as climate, precipitation and soil properties. Thus, this study cannot fully exclude the synergistic selective effect of abiotic factors on the differentiation of plant traits. However, the geographic gradient of herbivory pressure aligned closely with the observed divergence in defense traits, consistent with theoretical predictions. Quantitative defenses are maintained at high levels in regions with the specialist herbivore, but decline in regions where the specialist herbivore is absent, accompanied by increases in qualitative defenses and growth. The pattern aligned well with the predictions of plant defense theory. Although the relative contributions of herbivory, abiotic adaptation, founder effects and genetic drift cannot be fully disentangled based on the current common garden experiment, the observed defense-growth trade-off and trait divergence aligned with theoretical predictions of the revised EICA hypothesis, suggesting that herbivore mediated selection may be one important contributor to defense differentiation among *S. rostratum* populations. Moreover, classification of herbivore pressure is based on species occurrence rather than quantitative measures of herbivory intensity, population density, or temporal variation. Future studies incorporating multiple years of surveys, herbivore density quantification, and plant damage assessments will be needed to establish the relationship between selection strength and the magnitude of defense differentiation.

### 4.2 Bioassays on two types of defensive metabolites

As qualitative defensive metabolites, the alkaloids mainly exert their effects through acute toxicity and rapid anorexia (Rhoades and Cates, 1976). These compounds directly act on the physiological systems of insects, such as the nervous system and the digestive tract, and cause strong toxic reactions and feeding avoidance at low concentrations (Steppuhn et al., 2004). Therefore, they exhibit remarkably high antifeedant and growth inhibitory activities in short-term bioassays. The characteristic of low-dose requirement, high bioactivity and rapid onset is the hallmark of qualitative defensive metabolites. By contrast, phenolic acids, the typical quantitative defensive metabolites, mainly exert their effects through long-term digestive inhibition rather than acute poisoning (War et al., 2013). These compounds reduce the digestibility of food by forming complexes with nutrients in food, and lack potent toxicity themselves. Their defensive effects are cumulative: only after prolonged feeding, they have a significant impact on the adaptability of insects, such as retarded development and reduced body weight (Lv et al., 2025). In the present study, the biological assays were limited to the early larval stage, which is too short to fully reveal the long-term defensive effects of phenolic acids. It could explain why the bioactivity of phenolic acids was weaker than that of alkaloids in the short-term assays.

### 4.3 Adaptive defensive strategies driven by herbivory pressure

The content determination results showed that there were significant differences in the defensive metabolite profiles between the two geographical populations. The total content of qualitative defensive metabolites (alkaloids) was significantly higher in Liaoning populations, while the total content of quantitative defensive metabolites (phenolic acids) was significantly higher in Xinjiang populations. In both populations, the content of phenolic acids was generally higher than that of alkaloids (**Supplementary Figure 4**). From the perspective of the dose-dependent defense, quantitative defense exhibits strict concentration dependence. Only when a high concentration is reached can nutrients be effectively complexed and digestive enzyme activity be inhibited, thereby exerting a stable inhibitory effect on digestion (War et al., 2013; Lv et al., 2025). Therefore, plants need to maintain relatively high absolute concentrations to achieve their defensive functions. By contrast, qualitative defense is characterized by “low dose, high activity”. The compound only requires an extremely low concentration to target the physiological systems of specific insects and exert toxic effects. Therefore, the defense function can be achieved at a relatively lower concentration (Liu et al., 2024; Leite Dias and D'Auria, 2025).

From the perspective of the herbivory pressure faced by the population, the evolution of plant defense strategies has always been strongly influenced by the structure of the local herbivore community. The compositions of herbivory pressure are the key selective factors determining the allocation of defense types (Zhu et al., 2026). The Xinjiang populations are simultaneously subjected to dual herbivory pressures from the specialist herbivore and the generalist herbivore, resulting in more complex selective pressures. In this habitat, relying solely on alkaloid-based qualitative defense poses a relatively high evolutionary risk. Specialist herbivores may have the potential to rapidly evolve detoxification capabilities, while high-concentration phenolic acid-based quantitative defense has a more stable defensive effect and can be effective against both specialist and generalist herbivores. Therefore, the strategy becomes the key defense strategy of Xinjiang populations. Liaoning populations are only subjected to herbivory pressure from the generalist herbivores, and lack the selective pressure from specialist herbivores. In this situation, the low-cost and high-efficiency qualitative defense based on alkaloids could effectively deal with generalists and meet the basic defense requirements. As a result, the population gradually evolves into a “high qualitative defense, low quantitative defense” pattern.

### 4.4 Evolution of resource allocation under differential defense costs

The results of growth trait measurements and correlation analyses showed that all growth traits of Liaoning populations, including relative leaf chlorophyll content, maximum light-saturated photosynthetic rate, specific leaf area, biomass, plant height, basal diameter, and relative growth rate, were significantly higher than those of Xinjiang populations (**Figure 3**). Moreover, the contents of quantitative defensive metabolites were significantly negatively correlated with plant growth traits, while the contents of qualitative defensive metabolites were significantly positively correlated with growth traits (**Figure 5**). These results clearly demonstrated the difference in resource costs between the two defense types, confirmed the resource trade-off pattern underlying invasive plant defense evolution, and aligned well with the predictions of the growth-differentiation balance hypothesis (Herms and Mattson, 1992; Monson et al., 2021).

According to the defense cost theory, there is an inherent trade-off between plant defense and growth. Resources allocated to the synthesis of secondary metabolites cannot generally be used for growth and reproduction. This trade-off in resource allocation is the core driving force for the evolution of plant life history strategies (Bass, 2025). Among the two defense types, quantitative defenses require a high-concentration accumulation to exert their effects, resulting in a much higher total resource investment than low-abundance qualitative defensive metabolites. The substantial resources allocated to phenolic acid synthesis and accumulation inevitably reduce the resources available for growth and reproduction. Accordingly, Xinjiang populations maintained a relatively high level of quantitative defense, but the growth rate and biomass accumulation significantly declined. In contrast, qualitative defensive metabolites only require low-dose synthesis, and have a relatively weaker inhibitory effect on growth. Therefore, the Liaoning population was still able to maintain vigorous growth despite the high accumulation of alkaloids.

The growth-defense pattern also provided chemical ecological evidence for the revised EICA hypothesis. The classic EICA hypothesis posits that when invasive plants escape from their native specialist herbivores, they will reallocate the resources originally used for defense to growth and reproduction, thereby gaining a competitive advantage (Blossey and Nötzold, 1995). In fact, alien plants only escape from specialist herbivores in introduced ranges, and are still affected by generalist herbivores (Parker et al., 2006). Therefore, Müller-Schärer et al. (2004) proposed a revised EICA hypothesis based on the changes in the composition of enemies after the invasion of alien plants and the different defense mechanisms of alien plants against specialist and generalist herbivores. It suggests that alien plants may only reduce the defense against specialist herbivores, but not necessarily reduce the defense against generalist herbivores, and the change of defense strategy is mediated by chemical effects of secondary metabolites.

Most previous studies merely compared the differences in the degree of damage suffered by invasive plants in their native and introduced ranges by specialist herbivores, and then speculated on the evolution of defense abilities of invasive plants under different selection pressures of herbivores (Sun et al., 2023; Yin et al., 2023). A few studies only measured the differences in the contents of total alkaloids or phenols in invasive plants (Wang et al., 2012; Sakata et al., 2014; Wan et al., 2019). The study found that in habitats where the specialist herbivores were absent, Liaoning populations reduced the costly quantitative defense of phenolic acids and instead increased the low-cost qualitative defense of alkaloids. While ensuring that *S. rostratum* can defend against general herbivores, it released more resources for growth and reproduction, achieving the optimal allocation of resources. However, Xinjiang populations still faced the pressure from specialist herbivores and retained the high-input quantitative defense strategy, resulting in the restricted growth (**Figure 6**). It further verified the shaping effect of herbivory pressure on defense strategies.

**Figure 6:**
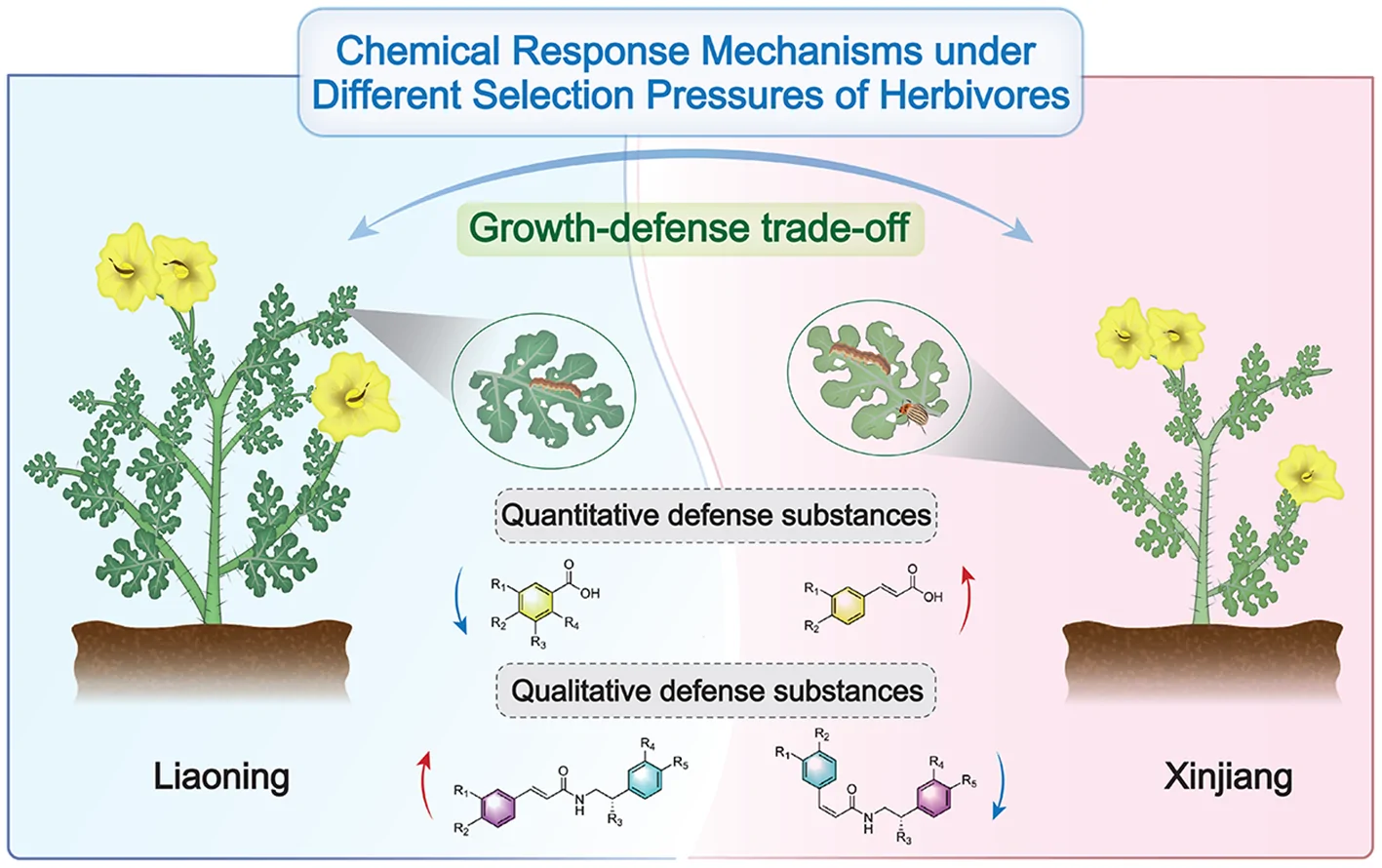
Conceptual model illustrating hypothesized resource-allocation shifts in defense strategies under different selection pressures of herbivores. This schematic represents a hypothetical evolutionary scenario and does not exclude contributions from founder effects, genetic drift or abiotic selection.

## 5 Conclusion

Adaptive evolutionary patterns of quantitative and qualitative defensive metabolites were systematically investigated in the invasive plant *S. rostratum* under different selection pressures of herbivores, integrating field surveys, common garden experiments, metabolite identification, and insect bioassays. Divergent defense traits were exhibited among *S. rostratum* populations under different selection pressures of herbivores: populations under combined pressure from specialist and generalist herbivores (Xinjiang) maintained high levels of costly quantitative defenses at the expense of plant growth, whereas populations experiencing only generalist herbivores (Liaoning) shifted resource allocation toward low-cost qualitative defenses and retained vigorous growth. This defense divergence reflected a clear growth-defense resource trade-off, consistent with core predictions of the growth-differentiation balance hypothesis. Metabolite-level chemical ecological evidence for the revised EICA hypothesis was generated, which advanced mechanistic understanding of plant invasion evolution and offered a chemical basis for targeted management of *S. rostratum* and other invasive weeds.

## Data Availability

The datasets presented in this study can be found in online repositories. The names of the repository/repositories and accession number(s) can be found below: http://doi.org/10.6084/m9.figshare.33102299.
