# Supplementary material for: Chemical response mechanisms of Solanum rostratum under different selection pressures of herbivores
Source: Front Plant Sci. 2026 Sep 30;17:1955283. doi: 10.3389/fpls.2026.1955283 (PMC13661573; doi:10.3389/fpls.2026.1955283)
Supplement: Supplementary file 1 [file DataSheet1.docx]

Supplementary Material

1 **Supplementary Figures**

**Supplementary Figure 1.** The wild growth conditions of *S. rostratum* in Liaoning and Xinjiang. (a)−(f) represented the wild growth conditions of different populations in Liaoning. (g)−(l) represented the wild growth conditions of different populations in Xinjiang.

**Supplementary Figure 2.** The existence of herbivores in field investigations. (a) *L. decemlineata* observed on *S. rostratum* in Xinjiang. (b) *H. armigera* observed on *S. rostratum* in Xinjiang. (c) *H. armigera* observed on *S. rostratum* in Liaoning.

**Supplementary Figure 3.** The extraction and separation process of compounds **1**−**31**.

**Supplementary Figure 4.** The differences in the contents of defensive metabolites in different populations of *S. rostratum* in Liaoning and Xinjiang. (a) The difference in the contents of qualitative defensive metabolites. (b) The difference in the contents of quantitative defensive metabolites. **p*<0.05, ***p*<0.01 and ****p*<0.001.

2 **Supplementary Tables**

**Supplementary Table 1.** Geographical location information of different populations of *S. rostratum* in Liaoning and Xinjiang.

| Population | Site | Latitude | Longitude | Elevation (m) | Herbivore pressure type |
| --- | --- | --- | --- | --- | --- |
| Liaoning populations | Qinghemen District, Fuxin City | 41°45′ N | 121°26′ E | 103 | Only generalist |
|  | Xihe District, Fuxin City | 42°01′ N | 121°40′ E | 157 | Only generalist |
|  | Yi County, Jinzhou City | 41°31′ N | 121°14′ E | 75 | Only generalist |
|  | Linghai City, Jinzhou City | 41°12′ N | 121°29′ E | 26 | Only generalist |
|  | Beipiao City, Chaoyang City | 41°45′ N | 120°47′ E | 131 | Only generalist |
|  | Shuangta District, Chaoyang City | 41°37′ N | 120°28′ E | 219 | Only generalist |
| Xinjiang populations | Urumqi County, Urumqi City | 43°27′ N | 87°15′ E | 1040 | Specialist + generalist |
|  | Shuimogou District, Urumqi City | 43°49′ N | 87°46′ E | 922 | Specialist + generalist |
|  | Midong District, Urumqi City | 43°57′ N | 87°46′ E | 775 | Specialist + generalist |
|  | Toutunhe District, Urumqi City | 43°58′ N | 87°24′ E | 544 | Specialist + generalist |
|  | Toksun County, Turpan City | 42°50′ N | 88°36′ E | -43 | Specialist + generalist |
|  | Sangong Town, Changji City | 43°55′ N | 87°12′ E | 709 | Specialist + generalist |

**Supplementary Table 2.** Standard-curve parameters for defensive metabolites.

| Compounds | $Regression equation$ | R^2^ |
| --- | --- | --- |
| **Comp. 1** | y = 141.58x - 116.65 | 0.9967 |
| **Comp. 2** | y = 121.58x - 175.71 | 0.9982 |
| **Comp. 3** | y = 135.94x + 119.14 | 0.9946 |
| **Comp. 4** | y = 122.34x - 251.21 | 0.9983 |
| **Comp. 5** | y = 154.73x + 9.0236 | 0.9989 |
| **Comp. 6** | y = 131.57x + 263.79 | 0.9978 |
| **Comp. 7** | y = 141.55x - 49.638 | 0.9998 |
| **Comp. 8** | y = 152.49x + 126.29 | 0.9983 |
| **Comp. 9** | y = 110.5x - 61.16 | 0.9985 |
| **Comp. 10** | y = 116.17x + 86.381 | 0.9987 |
| **Comp. 11** | y = 127.15x + 92.02 | 0.9999 |
| **Comp. 12** | y = 153.16x + 195.32 | 0.997 |
| **Comp. 13** | y = 103.66x + 300.77 | 0.9948 |
| **Comp. 14** | y = 125.59x - 161.65 | 0.9986 |
| **Comp. 15** | y = 167.12x - 66.294 | 0.9994 |
| **Comp. 16** | y = 152.71x + 43.946 | 0.9991 |
| **Comp. 17** | y = 138.58x + 175.77 | 0.9979 |
| **Comp. 18** | y = 161.35x - 145.85 | 0.9998 |
| **Comp. 19** | y = 162.13x - 268.91 | 0.9989 |
| **Comp. 20** | y = 118.82x + 87.729 | 0.998 |
| **Comp. 21** | y = 82.315x + 176.74 | 0.9989 |
| **Comp. 22** | y = 135.08x + 184.67 | 0.9986 |
| **Comp. 23** | y = 105.54x + 276.51 | 0.9943 |
| **Comp. 24** | y = 106.92x + 246.36 | 0.9953 |
| **Comp. 25** | y = 93.085x + 104.86 | 0.9994 |
| **Comp. 26** | y = 119.48x + 346.21 | 0.9903 |
| **Comp. 27** | y = 95.532x + 185.2 | 0.9989 |
| **Comp. 28** | y = 109.05x - 75.07 | 0.998 |
| **Comp. 29** | y = 105.677x + 126.11 | 0.9951 |
| **Comp. 30** | y = 91.308x + 207.44 | 0.9969 |
| **Comp. 31** | y = 128.01x - 128.99 | 0.9989 |

**Supplementary Table 3.** ^1^H (600 MHz) NMR data for compounds **1**−**4**.

| Position | compound **1** | compound **2** | compound **3** | compound **4** |
| --- | --- | --- | --- | --- |
| 1 |  |  |  |  |
| 2 | *δ* 7.13 (1H, d, *J* = 1.59 Hz) | *δ* 7.12 (1H, s) | *δ* 7.10 (1H, d, *J* = 1.88 Hz) | *δ* 7.49 (1H, d, *J* = 8.42 Hz) |
| 3 |  |  |  | *δ* 6.94 (1H, d, *J* = 8.42 Hz) |
| 4 |  |  |  |  |
| 5 | *δ* 6.80 (1H, dd, *J* = 1.59, 8.53 Hz) | *δ* 6.80 (1H, d, *J* = 8.27 Hz) | *δ* 6.79 (1H, d, *J* = 8.17 Hz) | *δ* 6.94 (1H, d, *J* = 8.42 Hz) |
| 6 | *δ* 7.03 (1H, dd, *J* = 1.59, 8.53 Hz) | *δ* 7.03 (1H, d, *J* = 8.27 Hz) | *δ* 7.02 (1H, dd, *J* = 1.88, 8.17 Hz) | *δ* 7.49 (1H, d, *J* = 8.42 Hz) |
| 7 | *δ* 7.45 (1H, d, *J* = 15.74 Hz) | *δ* 7.45 (1H, d, *J* = 15.72 Hz) | *δ* 7.44 (1H, d, *J* = 15.72 Hz) | *δ* 7.48 (1H, d, *J* = 15.67 Hz) |
| 8 | *δ* 6.48 (1H, d, *J* = 15.74 Hz) | *δ* 6.47 (1H, d, *J* = 15.72 Hz) | *δ* 6.40 (1H, d, *J* = 15.72 Hz) | *δ* 6.44 (1H, d, *J* = 15.67 Hz) |
| 9 |  |  |  |  |
| 1′ |  |  |  |  |
| 2′ | *δ* 7.17 (1H, d, *J* = 8.53 Hz) | *δ* 7.23 (1H, d, *J* = 8.45 Hz) | *δ* 7.06 (1H, dd, *J* = 1.95, 6.48 Hz) | *δ* 7.06 (1H, d, *J* = 8.11 Hz) |
| 3′ | *δ* 6.80 (1H, dd, *J* = 1.59, 8.53 Hz) | *δ* 6.77 (1H, d, *J* = 8.45 Hz) | *δ* 6.72 (1H, dd, *J* = 1.95, 6.48 Hz) | *δ* 6.72 (1H, d, *J* = 8.11 Hz) |
| 4′ |  |  |  |  |
| 5′ | *δ* 6.80 (1H, dd, *J* = 1.59, 8.53 Hz) | *δ* 6.77 (1H, d, *J* = 8.45 Hz) | *δ* 6.72 (1H, dd, *J* = 1.95, 6.48 Hz) | *δ* 6.72 (1H, d, *J* = 8.11 Hz) |
| 6′ | *δ* 7.17 (1H, d, *J* = 8.53 Hz) | *δ* 7.23 (1H, d, *J* = 8.45 Hz) | *δ* 7.06 (1H, dd, *J* = 1.95, 6.48 Hz) | *δ* 7.06 (1H, d, *J* = 8.11 Hz) |
| 7′ | *δ* 4.25 (1H, br s) | *δ* 4.72 (1H, br s) | *δ* 3.46 (2H, t, *J* = 7.56 Hz) | *δ* 2.75 (2H, t, *J* = 7.56 Hz) |
| 8′ | *δ* 3.40 (1H, dd, *J* = 4.54, 13.80 Hz)  *δ* 3.52 (1H, dd, *J* = 8.53, 13.80 Hz) | *δ* 3.44 (1H, br s)  *δ* 3.53 (1H, dd, *J* = 4.74, 13.65 Hz) | *δ* 2.75 (2H, t, *J* = 7.56 Hz) | *δ* 3.46 (2H, t, *J* = 7.56 Hz) |
| 3-OCH_3_ | *δ* 3.88 (3H, s) | *δ* 3.88 (3H, s) |  |  |
| 4-OCH_3_ |  |  |  | *δ* 3.82 (3H, s) |
| 7'-OCH_3_ | *δ* 3.21 (3H, s) |  |  |  |

**Supplementary Table 4.** ^1^H (600 MHz) NMR data for compounds **5**−**8**.

| Position | compound **5** | compound **6** | compound **7** | compound **8** |
| --- | --- | --- | --- | --- |
| 1 |  |  |  |  |
| 2 | *δ* 7.40 (1H, d, *J* = 8.56 Hz) | *δ* 7.40 (1H, d, *J* = 8.50 Hz) | *δ* 7.40 (1H, d, *J* = 8.39 Hz) | *δ* 7.40 (1H, d, *J* = 8.20 Hz) |
| 3 | *δ* 6.79 (1H, d, *J* = 8.56 Hz) | *δ* 6.78 (1H, t, *J* = 9.18 Hz) | *δ* 6.79 (1H, d, *J* = 8.39 Hz) | *δ* 6.78 (1H, dd, *J* = 5.47, 8.20 Hz) |
| 4 |  |  |  |  |
| 5 | *δ* 6.79 (1H, d, *J* = 8.56 Hz) | *δ* 6.78 (1H, t, *J* = 9.18 Hz) | *δ* 6.79 (1H, d, *J* = 8.39 Hz) | *δ* 6.78 (1H, dd, *J* = 5.47, 8.20 Hz) |
| 6 | *δ* 7.40 (1H, d, *J* = 8.56 Hz) | *δ* 7.40 (1H, d, *J* = 8.50 Hz) | *δ* 7.40 (1H, d, *J* = 8.39 Hz) | *δ* 7.40 (1H, d, *J* = 8.20 Hz) |
| 7 | *δ* 7.46 (1H, d, *J* = 15.68 Hz) | *δ* 7.45 (1H, d, *J* = 15.72 Hz) | *δ* 7.46 (1H, d, *J* = 15.56 Hz) | *δ* 7.46 (1H, d, *J* = 15.77 Hz) |
| 8 | *δ* 6.45 (1H, d, *J* = 15.68 Hz) | *δ* 6.44 (1H, d, *J* = 15.72 Hz) | *δ* 6.45 (1H, d, *J* = 15.56 Hz) | *δ* 6.44 (1H, d, *J* = 15.77 Hz) |
| 9 |  |  |  |  |
| 1′ |  |  |  |  |
| 2′ | *δ* 6.79 (2H, d, *J* = 8.56 Hz) | *δ* 7.17 (1H, d, *J* = 8.37 Hz) | *δ* 6.75 (1H, dd, *J* = 8.01, 19.73 Hz) | *δ* 7.22 (1H, d, *J* = 8.45 Hz) |
| 3′ |  | *δ* 6.78 (1H, t, *J* = 9.18 Hz) |  | *δ* 6.78 (1H, dd, *J* = 5.47, 8.45 Hz) |
| 4′ |  |  |  |  |
| 5′ | *δ* 6.77 (1H, d, *J* = 8.18 Hz) | *δ* 6.78 (1H, t, *J* = 9.18 Hz) | *δ* 6.85 (1H, s) | *δ* 6.78 (1H, dd, *J* = 5.47, 8.45 Hz) |
| 6′ | *δ* 6.67 (1H, dd, *J* = 1.76, 8.18 Hz) | *δ* 7.17 (1H, d, *J* = 8.37 Hz) | *δ* 6.75 (1H, dd, *J* = 8.01, 19.73 Hz) | *δ* 7.22 (1H, d, *J* = 8.45 Hz) |
| 7′ | *δ* 4.18 (1H, br s) | *δ* 4.24 (1H, br s) | *δ* 4.65 (1H, br s) | *δ* 4.72 (1H, br s) |
| 8′ | *δ* 3.46 (2H, s) | *δ* 3.46 (1H, s)  *δ* 3.51 (1H, dd, *J* = 4.56, 13.87 Hz) | *δ* 3.42 (1H, s)  *δ* 3.52 (1H, dd, *J* = 4.86, 13.65 Hz) | *δ* 3.46 (1H, s)  *δ* 3.53 (1H, dd, *J* = 4.82, 13.62 Hz) |
| 3-OCH3 |  |  |  |  |
| 7'-OCH3 | *δ* 3.22 (3H, s) | *δ* 3.22 (3H, s) |  |  |

**Supplementary Table 5.** ^1^H (600 MHz) NMR data for compounds **9**−**12**.

| Position | compound **9** | compound **10** | compound **11** | compound **12** |
| --- | --- | --- | --- | --- |
| 1 |  |  |  |  |
| 2 | *δ* 7.38 (1H, d, *J* = 8.30 Hz) | *δ* 7.39 (1H, dd, *J* = 1.98, 6.69 Hz) | *δ* 7.35 (1H, d, *J* = 1.94 Hz) | *δ* 7.14 (1H, d, *J* = 8.53 Hz) |
| 3 | *δ* 6.77 (1H, d, *J* = 8.30 Hz) | *δ* 6.78 (1H, dd, *J* = 1.98, 6.69 Hz) |  | *δ* 6.74 (1H, d, *J* = 8.53 Hz) |
| 4 |  |  |  |  |
| 5 | *δ* 6.77 (1H, d, *J* = 8.30 Hz) | *δ* 6.78 (1H, dd, *J* = 1.98, 6.69 Hz) | *δ* 6.73 (1H, d, *J* = 8.25 Hz) | *δ* 6.74 (1H, d, *J* = 8.53 Hz) |
| 6 | *δ* 7.38 (1H, d, *J* = 8.30 Hz) | *δ* 7.39 (1H, dd, *J* = 1.98, 6.69 Hz) | *δ* 6.92 (1H, dd, *J* = 1.94, 8.25 Hz) | *δ* 7.14 (1H, d, *J* = 8.53 Hz) |
| 7 | *δ* 7.44 (1H, d, *J* = 15.68 Hz) | *δ* 7.45 (1H, d, *J* = 15.72 Hz) | *δ* 6.61 (1H, d, *J* = 12.85 Hz) | *δ* 5.83 (1H, d, *J* = 12.61 Hz) |
| 8 | *δ* 6.39 (1H, d, *J* = 15.68 Hz) | *δ* 6.38 (1H, d, *J* = 15.72 Hz) | *δ* 5.81 (1H, d, *J* = 12.85 Hz) | *δ* 6.63 (1H, d, *J* = 12.61 Hz) |
| 9 |  |  |  |  |
| 1′ |  |  |  |  |
| 2′ | *δ* 6.67 (1H, br s) | *δ* 7.06 (1H, dd, *J* = 2.03, 6.42 Hz) | *δ* 6.98 (1H, dd, *J* = 1.98, 6.63 Hz) | *δ* 7.39 (1H, d, *J* = 8.76 Hz) |
| 3′ |  | *δ* 6.72 (1H, dd, *J* = 2.03, 6.42 Hz) | *δ* 6.69 (1H, dd, *J* = 1.98, 6.63 Hz) | *δ* 6.78 (1H, d, *J* = 8.76 Hz) |
| 4′ |  |  |  |  |
| 5′ | *δ* 6.70 (1H, d, *J* = 8.10 Hz) | *δ* 6.72 (1H, dd, *J* = 2.03, 6.42 Hz) | *δ* 6.69 (1H, dd, *J* = 1.98, 6.63 Hz) | *δ* 6.78 (1H, d, *J* = 8.76 Hz) |
| 6′ | *δ* 6.54 (1H, dd, *J* = 8.10, 1.57 Hz) | *δ* 7.05 (1H, dd, *J* = 2.03, 6.42 Hz) | *δ* 6.98 (1H, dd, *J* = 1.98, 6.63 Hz) | *δ* 7.39 (1H, d, *J* = 8.76 Hz) |
| 7′ | *δ* 2.69 (2H, t, *J* = 7.28 Hz) | *δ* 3.45 (2H, t, *J* = 7.25 Hz) | *δ* 2.70 (2H, t, *J* = 7.25 Hz) | *δ* 4.20 (1H, br s) |
| 8′ | *δ* 3.44 (2H, t, *J* = 7.28 Hz) | *δ* 2.76 (2H, t, *J* = 7.25 Hz) | *δ* 3.40 (2H, br s) | *δ* 3.34 (1H, br s)  *δ* 3.45 (1H, dd, *J* = 4.50, 13.75 Hz) |
| 3-OCH3 |  |  | *δ* 3.82 (3H, s) |  |
| 7'-OCH3 |  |  |  | *δ* 3.15 (3H, s) |

**Supplementary Table 6.** ^1^H (600 MHz) NMR data for compounds **13**−**15**.

| Position | compound **13** | compound **14** | compound **15** |
| --- | --- | --- | --- |
| 1 |  |  |  |
| 2 | *δ* 7.39 (1H, d, *J* = 8.57 Hz) | *δ* 7.18 (1H, d, *J* = 8.40 Hz) | *δ* 7.36 (1H, dd, *J* = 1.74, 6.56 Hz) |
| 3 | *δ* 6.72 (1H, d, *J* = 4.16 Hz) | *δ* 6.71 (1H, d, *J* = 8.40 Hz) | *δ* 6.72 (1H, dd, *J* = 6.56, 1.74 Hz) |
| 4 |  |  |  |
| 5 | *δ* 6.72 (1H, d, *J* = 4.16 Hz) | *δ* 6.71 (1H, d, *J* = 8.40 Hz) | *δ* 6.72 (1H, dd, *J* = 6.56, 1.74 Hz) |
| 6 | *δ* 7.39 (1H, d, *J* = 8.57 Hz) | *δ* 7.18 (1H, d, *J* = 8.40 Hz) | *δ* 7.36 (1H, dd, *J* = 1.74, 6.56 Hz) |
| 7 | *δ* 6.62 (1H, d, *J* = 12.73 Hz) | *δ* 5.81 (1H, d, *J* = 12.65 Hz) | *δ* 6.61 (1H, d, *J* = 12.66 Hz) |
| 8 | *δ* 5.82 (1H, d, *J* = 12.73 Hz) | *δ* 6.62 (1H, d, *J* = 12.65 Hz) | *δ* 5.79 (1H, d, *J* = 12.66 Hz) |
| 9 |  |  |  |
| 1′ |  |  |  |
| 2′ | *δ* 6.73 (1H, d, *J* = 3.72 Hz) | *δ* 7.38 (1H, d, *J* = 8.52 Hz) | *δ* 7.01 (1H, dd, *J* = 6.74, 2.00 Hz) |
| 3′ |  | *δ* 6.75 (1H, d, *J* = 8.52 Hz) | *δ* 6.70 (1H, dd, *J* = 6.74, 2.00 Hz) |
| 4′ |  |  |  |
| 5′ | *δ* 6.68 (1H, dd, *J* = 1.62, 8.20 Hz) | *δ* 6.75 (1H, d, *J* = 8.52 Hz) | *δ* 6.70 (1H, dd, *J* = 6.74, 2.00 Hz) |
| 6′ | *δ* 6.83 (1H, s) | *δ* 7.38 (1H, d, *J* = 8.52 Hz) | *δ* 7.01 (1H, dd, *J* = 6.74, 2.00 Hz) |
| 7′ | *δ* 4.62 (1H, s) | *δ* 3.46 (1H, dd, *J* = 13.52, 5.20 Hz)  *δ* 4.67 (1H, dd, *J* = 7.62, 5.20 Hz) | *δ* 2.69 (2H, t, *J* = 7.35 Hz) |
| 8′ | *δ* 3.46 (1H, s) | *δ* 3.37 (1H, dd, *J* = 13.52, 7.62 Hz) | *δ* 3.38 (2H, br s) |

**Supplementary Table 7.** ^1^H (600 MHz) NMR data for compounds **16**−**18**.

| Position | compound **16** | compound **17** | compound **18** |
| --- | --- | --- | --- |
| 1 |  |  |  |
| 2 |  | *δ* 8.30 (1H, s) |  |
| 3 |  |  |  |
| 4 | *δ* 4.50 (1H, d, *J* = 9.91 Hz) |  |  |
| 5 | *δ* 3.90 (1H, dd, *J* = 9.91, 8.22 Hz)  *δ* 3.21 (1H, dd, *J* = 9.91, 1.65 Hz) |  |  |
| 6 | *δ* 7.38 (1H, d, *J* = 1.65 Hz) |  | *δ* 7.81 (1H, s) |
| 7 |  |  | *δ* 1.88 (3H, s) |
| 8 |  | *δ* 8.18 (1H, s) |  |
| 1′ |  | *δ* 5.96 (1H, d, *J* = 5.98 Hz) | *δ* 6.28 (1H, t, *J* = 6.65 Hz) |
| 2′ | *δ* 7.19 (1H, d, *J* = 8.58 Hz) | *δ* 4.73 (1H, t, *J* = 5.42 Hz) | *δ* 2.23 (2H, br s) |
| 3′ | *δ* 6.61 (1H, d, *J* = 8.58 Hz) | *δ* 4.32 (1H, dd, *J* = 4.99, 2.43 Hz) | *δ* 4.39 (1H, br s) |
| 4′ |  | *δ* 4.16 (1H, q, *J* = 2.50 Hz) | *δ* 3.90 (1H, br s) |
| 5′ | *δ* 6.61 (1H, d, *J* = 8.58 Hz) | *δ* 3.73 (1H, dd, *J* = 12.61, 2.59 Hz)  *δ* 3.89 (1H, dd, *J* = 12.61, 2.37 Hz) | *δ* 3.74 (1H, dd, *J* = 12.11, 3.58 Hz)  *δ* 3.80 (1H, dd, *J* = 12.07, 3.14 Hz) |
| 6′ | *δ* 7.19 (1H, d, *J* = 8.58 Hz) |  |  |
| 1′′ |  |  |  |
| 2′′ | *δ* 7.05 (1H, d, *J* = 8.31 Hz) |  |  |
| 3′′ | *δ* 6.70 (1H, d, *J* = 8.31 Hz) |  |  |
| 4′′ |  |  |  |
| 5′′ | *δ* 6.70 (1H, d, *J* = 8.31 Hz) |  |  |
| 6′′ | *δ* 7.06 (1H, d, *J* = 8.31 Hz) |  |  |

**Supplementary Table 8.** ^1^H (600 MHz) NMR data for compounds **19**−**22**.

| Position | compound **19** | compound **20** | compound **21** | compound **22** |
| --- | --- | --- | --- | --- |
| 1 |  |  |  |  |
| 2 | *δ* 7.55 (1H, br s) | *δ* 7.06 (2H, s) | *δ* 7.43 (1H, d, *J* = 12.13 Hz) | *δ* 7.88 (1H, d, *J* = 8.55 Hz) |
| 3 | *δ* 6.84 (1H, d, *J* = 8.75 Hz) |  | *δ* 6.80 (1H, d, *J* = 8.11 Hz) | *δ* 6.82 (1H, d, *J* = 8.55 Hz) |
| 4 |  |  |  |  |
| 5 |  |  |  | *δ* 6.82 (1H, d, *J* = 8.55 Hz) |
| 6 | *δ* 7.55 (1H, br s) |  | *δ* 7.43 (1H, d, *J* = 12.13 Hz) | *δ* 7.88 (1H, d, *J* = 8.55 Hz) |
| 7 |  |  |  |  |
| 5-OCH_3_ | *δ* 3.88 (3H, s) |  |  |  |

**Supplementary Table 9.** ^1^H (600 MHz) NMR data for compounds **23**−**27**.

| Position | compound **23** | compound **24** | compound **25** | compound **26** | compound **27** |
| --- | --- | --- | --- | --- | --- |
| 1 |  |  |  |  |  |
| 2 | *δ* 6.94 (1H, d, *J* = 2.00 Hz) | *δ* 7.88 (1H, d, *J* = 8.55 Hz) | *δ* 7.43 (1H, t, *J* = 2.10 Hz) |  | *δ* 7.16 (1H, s) |
| 3 |  | *δ* 6.82 (1H, d, *J* = 8.55 Hz) |  | *δ* 6.88 (1H, br s) |  |
| 4 | *δ* 6.46 (1H, br s) |  | *δ* 6.99 (1H, q, *J* = 8.05 Hz) | *δ* 7.42 (1H, t, *J* = 7.80 Hz) |  |
| 5 |  | *δ* 6.82 (1H, d, *J* = 8.55 Hz) | *δ* 7.49 (1H, d, *J* = 7.63 Hz) | *δ* 6.85 (1H, br s) | *δ* 6.81 (1H, d, *J* = 8.16 Hz) |
| 6 | *δ* 6.94 (1H, d, *J* = 2.00 Hz) | *δ* 7.88 (1H, d, *J* = 8.55 Hz) | *δ* 7.26 (1H, t, *J* = 7.81 Hz) | *δ* 7.84 (1H, d, *J* = 8.00 Hz) | *δ* 7.05 (1H, d, *J* = 8.16 Hz) |
| 7 |  |  |  |  | *δ* 7.60 (1H, d, *J* = 15.88 Hz) |
| 8 |  |  |  |  | *δ* 6.31 (1H, d, *J* = 15.88 Hz) |
| 3-OCH_3_ |  |  |  |  | *δ* 3.88 (3H, s) |

**Supplementary Table 10.** ^1^H (600 MHz) NMR data for compounds **28**−**31**.

| Position | compound **28** | compound **29** | compound **30** | compound **31** |
| --- | --- | --- | --- | --- |
| 1 |  |  |  |  |
| 2 | *δ* 7.03 (1H, s) | *δ* 7.20 (1H, t, *J* = 7.90 Hz) | *δ* 7.45 (1H, d, *J* = 8.52 Hz) | *δ* 6.58 (1H, d, *J* = 8.04 Hz) |
| 3 |  |  | *δ* 6.81 (1H, d, *J* = 8.52 Hz) | *δ* 6.70 (1H, d, *J* = 8.04 Hz) |
| 4 |  | *δ* 6.99 (1H, br s) |  |  |
| 5 | *δ* 6.78 (1H, d, *J* = 8.27 Hz) | *δ* 6.83 (1H, dd, *J* = 1.84, 8.17 Hz) | *δ* 6.81 (1H, d, *J* = 8.52 Hz) |  |
| 6 | *δ* 6.93 (1H, d, *J* = 8.27 Hz) | *δ* 7.04 (1H, d, *J* = 7.65 Hz) | *δ* 7.45 (1H, d, *J* = 8.52 Hz) | *δ* 6.72 (1H, s) |
| 7 | *δ* 7.53 (1H, d, *J* = 16.05 Hz) | *δ* 7.59 (1H, d, *J* = 15.96 Hz) | *δ* 7.60 (1H, d, *J* = 15.92 Hz) | *δ* 3.41 (2H, s) |
| 8 | *δ* 6.23 (1H, d, *J* = 16.05 Hz) | *δ* 6.41 (1H, d, *J* = 15.96 Hz) | *δ* 6.29 (1H, d, *J* = 15.92 Hz) |  |

**Supplementary Table 11.** ^13^C (150 MHz) NMR data for compounds **1**−**9**.

| Position | compound **1** | compound **2** | compound **3** | compound **4** | compound **5** | compound **6** | compound **7** | compound **8** | compound **9** |
| --- | --- | --- | --- | --- | --- | --- | --- | --- | --- |
| 1 | *δ* 128.2 | *δ* 128.2 | *δ* 127.9 | *δ* 128.8 | *δ* 127.6 | *δ* 127.2 | *δ* 127.6 | *δ* 127.4 | *δ* 127.6 |
| 2 | *δ* 111.5 | *δ* 111.5 | *δ* 111.4 | *δ* 130.4 | *δ* 130.5 | *δ* 130.6 | *δ* 130.5 | *δ* 130.6 | *δ* 130.5 |
| 3 | *δ* 149.3 | *δ* 149.8 | *δ* 150.4 | *δ* 115.3 | *δ* 116.7 | *δ* 116.9 | *δ* 116.7 | *δ* 116.8 | *δ* 116.6 |
| 4 | *δ* 149.9 | *δ* 149.2 | *δ* 149.4 | *δ* 162.5 | *δ* 160.7 | *δ* 158.6 | *δ* 160.6 | *δ* 158.1 | *δ* 160.4 |
| 5 | *δ* 116.4 | *δ* 116.4 | *δ* 116.6 | *δ* 115.3 | *δ* 116.7 | *δ* 116.9 | *δ* 116.7 | *δ* 116.8 | *δ* 116.6 |
| 6 | *δ* 123.3 | *δ* 123.2 | *δ* 123.3 | *δ* 130.3 | *δ* 130.5 | *δ* 130.6 | *δ* 130.6 | *δ* 130.6 | *δ* 130.5 |
| 7 | *δ* 142.2 | *δ* 142.2 | *δ* 142.1 | *δ* 141.4 | *δ* 141.9 | *δ* 142.1 | *δ* 142.0 | *δ* 142.0 | *δ* 141.7 |
| 8 | *δ* 118.6 | *δ* 118.6 | *δ* 118.4 | *δ* 119.3 | *δ* 118.2 | *δ* 117.9 | *δ* 118.7 | *δ* 118.0 | *δ* 118.4 |
| 9 | *δ* 169.2 | *δ* 169.4 | *δ* 169.2 | *δ* 169.0 | *δ* 169.3 | *δ* 169.3 | *δ* 169.5 | *δ* 169.6 | *δ* 169.2 |
| 1′ | *δ* 131.4 | *δ* 134.7 | *δ* 131.3 | *δ* 131.2 | *δ* 132.2 | *δ* 131.3 | *δ* 135.5 | *δ* 134.7 | *δ* 132.0 |
| 2′ | *δ* 129.2 | *δ* 128.4 | *δ* 130.7 | *δ* 130.7 | *δ* 114.7 | *δ* 129.2 | *δ* 114.4 | *δ* 128.4 | *δ* 116.8 |
| 3′ | *δ* 116.3 | *δ* 116.1 | *δ* 116.2 | *δ* 116.2 | *δ* 146.4 | *δ* 116.3 | *δ* 145.9 | *δ* 116.1 | *δ* 146.2 |
| 4′ | *δ* 158.5 | *δ* 158.0 | *δ* 156.9 | *δ* 156.9 | *δ* 146.6 | *δ* 161.4 | *δ* 146.3 | *δ* 161.0 | *δ* 144.7 |
| 5′ | *δ* 116.3 | *δ* 116.1 | *δ* 116.2 | *δ* 116.2 | *δ* 116.2 | *δ* 116.3 | *δ* 116.1 | *δ* 116.1 | *δ* 116.3 |
| 6′ | *δ* 129.2 | *δ*128.4 | *δ*130.7 | *δ* 130.7 | *δ* 119.6 | *δ* 129.1 | *δ* 118.2 | *δ* 128.4 | *δ* 121.0 |
| 7′ | *δ* 83.2 | *δ*73.4 | *δ*35.8 | *δ* 35.7 | *δ* 83.3 | *δ* 83.2 | *δ* 73.5 | *δ* 73.4 | *δ* 36.0 |
| 8′ | *δ* 47.0 | *δ* 49.8 | *δ* 42.5 | *δ* 42.5 | *δ* 47.1 | *δ* 47.0 |  |  | *δ* 42.5 |
| 3-OCH_3_ | *δ* 56.3 | *δ* 56.3 | *δ* 56.3 |  |  |  |  |  |  |
| 4-OCH_3_ |  |  |  | *δ* 55.8 |  |  |  |  |  |
| 7'-OCH_3_ | *δ* 56.7 |  |  |  | *δ* 56.7 | *δ* 56.7 |  |  |  |

**Supplementary Table 12.** ^13^C (150 MHz) NMR data for compounds **10**−**15**, **17**, **18**.

| Position | compound **10** | compound **11** | compound **12** | compound **13** | compound **14** | compound **15** | compound **17** | compound **18** |
| --- | --- | --- | --- | --- | --- | --- | --- | --- |
| 1 | *δ* 127.2 | *δ* 128.3 | *δ* 127.9 | *δ* 127.9 | *δ* 127.8 | *δ* 127.8 |  |  |
| 2 | *δ* 130.5 | *δ* 113.9 | *δ* 132.3 | *δ* 132.4 | *δ* 132.4 | *δ* 130.6 | *δ* 153.4 | *δ* 166.5 |
| 3 | *δ* 116.0 | *δ* 148.9 | *δ* 115.9 | *δ* 115.9 | *δ* 116.1 | *δ* 116.2 |  |  |
| 4 | *δ* 156.9 | *δ* 148.6 | *δ* 159.2 | *δ* 159.2 | *δ* 159.5 | *δ* 159.5 | *δ* 150.0 | *δ* 152.4 |
| 5 | *δ* 116.0 | *δ* 115.9 | *δ* 115.9 | *δ* 116.0 | *δ* 116.1 | *δ* 116.2 | *δ* 130.8 | *δ* 111.5 |
| 6 | *δ* 130.5 | *δ* 124.8 | *δ* 131.3 | *δ* 132.4 | *δ* 132.4 | *δ* 130.7 | *δ* 157.6 | *δ* 138.1 |
| 7 | *δ* 141.9 | *δ* 138.4 | *δ* 138.3 | *δ* 138.6 | *δ* 138.6 | *δ* 138.0 |  | *δ* 12.4 |
| 8 | *δ* 118.0 | *δ* 121.4 | *δ* 121.3 | *δ* 121.1 | *δ* 121.0 | *δ* 121.3 | *δ* 142.0 |  |
| 9 | *δ* 169.3 | *δ* 170.3 | *δ* 170.2 | *δ* 170.4 | *δ* 170.4 | *δ* 170.3 |  |  |
| 1′ | *δ* 131.1 | *δ* 131.1 | *δ* 130.9 | *δ* 135.3 | *δ* 134.5 | *δ* 131.1 | *δ* 91.2 | *δ* 86.2 |
| 2′ | *δ* 130.7 | *δ* 130.6 | *δ* 129.2 | *δ* 114.3 | *δ* 128.5 | *δ* 132.2 | *δ* 75.4 | *δ* 41.1 |
| 3′ | *δ* 116.2 | *δ* 116.2 | *δ* 116.3 | *δ* 145.9 | *δ* 116.0 | *δ* 116.1 | *δ* 72.6 | *δ* 72.2 |
| 4′ | *δ* 159.5 | *δ* 156.9 | *δ* 158.5 | *δ* 146.3 | *δ* 158.1 | *δ* 156.9 | *δ* 88.2 | *δ* 88.8 |
| 5′ | *δ* 116.2 | *δ* 116.2 | *δ* 116.3 | *δ* 116.1 | *δ* 116.0 | *δ* 116.0 | *δ* 63.4 | *δ* 62.8 |
| 6′ | *δ* 130.7 | *δ* 130.7 | *δ* 129.2 | *δ* 118.7 | *δ* 128.5 | *δ* 132.2 |  |  |
| 7′ | *δ* 35.8 | *δ* 35.5 | *δ* 83.0 | *δ* 73.4 | *δ* 73.3 | *δ* 35.4 |  |  |
| 8′ | *δ* 42.5 | *δ* 42.3 | *δ* 46.7 | *δ* 48.0 | *δ* 47.9 | *δ* 42.2 |  |  |
| 3-OCH_3_ |  | *δ* 56.3 |  |  |  |  |  |  |
| 7'-OCH_3_ |  |  | *δ* 56.7 |  |  |  |  |  |

**Supplementary Table 13.** ^13^C (150 MHz) NMR data for compounds **16**, **21**, **22**.

| Position | compound **16** | compound **21** | compound **22** |
| --- | --- | --- | --- |
| 1 |  | *δ* 123.3 | *δ* 122.8 |
| 2 | *δ* 175.3 | *δ* 123.8 | *δ* 132.9 |
| 3 | *δ* 130.6 | *δ* 117.7 | *δ* 115.7 |
| 4 | *δ* 43.7 | *δ* 151.4 | *δ* 163.2 |
| 5 | *δ* 51.1 | *δ* 146.0 | *δ* 116.0 |
| 6 | *δ* 134.2 | *δ* 115.7 | *δ* 132.9 |
| 7 |  | *δ* 170.4 | *δ* 170.2 |
| 1′ | *δ* 126.4 |  |  |
| 2′ | *δ* 133.3 |  |  |
| 3′ | *δ* 116.9 |  |  |
| 4′ | *δ* 161.5 |  |  |
| 5′ | *δ* 116.9 |  |  |
| 6′ | *δ* 133.3 |  |  |
| 1′′ | *δ* 135.1 |  |  |
| 2′′ | *δ* 129.0 |  |  |
| 3′′ | *δ* 116.7 |  |  |
| 4′′ | *δ* 157.5 |  |  |
| 5′′ | *δ* 116.7 |  |  |
| 6′′ | *δ* 129.0 |  |  |

**Supplemsentary Table 14.** Specific rotation of compounds (${[\alpha]}_{D}^{25}$: specific rotation; c: concentration; CH_3_OH: solvent).

| Compounds | ${[\alpha]}_{D}^{25}$ (c 0.15, CH_3_OH) |
| --- | --- |
| **Comp. 1** | -13.30 |
| **Comp. 2** | -35.23 |
| **Comp. 5** | -6.40 |
| **Comp. 6** | -5.85 |
| **Comp. 7** | +25.70 |
| **Comp. 8** | -32.42 |
| **Comp. 12** | -5.32 |
| **Comp. 13** | +11.84 |
| **Comp. 14** | -21.40 |
| **Comp. 16** | +13.46 |

**Supplementary Table 15.** The results of the linear mixed model (LMM) of the effects of *S. rostratum* at different sites on its growth (In this model, we took site as the fixed factor and the population nested within site as the random effect. SPAD: leaf relative chlorophyll content; P_max_: maximum light-saturated photosynthetic rate; SLA: specific leaf area; AB: aboveground biomass; RB: root biomass; CW: crown width; H: plant height; SD: basal diameter of the stem; TB: total biomass; RGR: average relative growth rate).

|  | Site  (Xinjiang *vs* Liaoning) | | | | |
| --- | --- | --- | --- | --- | --- |
| Growth traits | *df* | t | *p* | Random population variance | Residual variance |
| SPAD | 10 | -3.57 | <0.01 | 7.481 | 34.800 |
| P_max_ | 10 | -9.48 | <0.001 | 2.689 | 39.482 |
| SLA | 10 | -4.93 | <0.001 | 147.000 | 299.800 |
| AB | 10 | -11.46 | <0.001 | 0.4553 | 2.4900 |
| RB | 10 | -13.10 | <0.001 | 0.0000 | 0.7442 |
| CW | 10 | -22.42 | <0.001 | 0.3730 | 8.5800 |
| H | 10 | -4.56 | <0.01 | 3.1540 | 9.2290 |
| SD | 10 | -14.10 | <0.001 | 0.02964 | 0.51454 |
| TB | 10 | -10.72 | <0.001 | 0.5487 | 3.3137 |
| RGR | 10 | -5.61 | <0.001 | 0.1319 | 0.4374 |

**Supplementary Table 16.** The results of the general linear model (GLM) of the effects of ethanol extracts of *S. rostratum* from different sites on herbivores (AEL: antifeedant activities of ethanol extracts against *L. decemlineata*; GEL: growth inhibition activities of ethanol extracts against *L. decemlineata*; AEH: antifeedant activities of ethanol extracts against *H. armigera*; GEH: growth inhibition activities of ethanol extracts against *H. armigera*).

|  | Site  (Xinjiang *vs* Liaoning) | | |
| --- | --- | --- | --- |
| Defense | *df* | t | *p* |
| AEL | 10 | 5.78 | <0.001 |
| GEL | 10 | 6.03 | <0.001 |
| AEH | 10 | -8.76 | <0.001 |
| GEH | 10 | -3.30 | <0.01 |

**sSupplementary Table 17.** The results of the general linear model (GLM) of the effects of different sites on the contents of defensive metabolites in *S. rostratum*.

|  | Site  (Xinjiang *vs* Liaoning) | | |
| --- | --- | --- | --- |
| Compounds | *df* | t | *p* |
| **Comp. 2** | 10 | -2.42 | <0.05 |
| **Comp. 3** | 10 | -2.92 | <0.05 |
| **Comp. 4** | 10 | -6.65 | <0.001 |
| **Comp. 5** | 10 | -1.74 |  |
| **Comp. 6** | 10 | -2.49 | <0.05 |
| **Comp. 7** | 10 | -2.00 |  |
| **Comp. 8** | 10 | -0.91 |  |
| **Comp. 9** | 10 | -1.32 |  |
| **Comp. 10** | 10 | -5.61 | <0.001 |
| **Comp. 11** | 10 | -1.90 |  |
| **Comp. 12** | 10 | -2.86 | <0.05 |
| **Comp. 13** | 10 | -2.47 | <0.05 |
| **Comp. 14** | 10 | -1.61 |  |
| **Comp. 15** | 10 | -1.46 |  |
| **Comp. 17** | 10 | -0.45 |  |
| **Comp. 18** | 10 | 0.09 |  |
| **Comp. 19** | 10 | 3.74 | <0.01 |
| **Comp. 20** | 10 | 2.24 | <0.05 |
| **Comp. 21** | 10 | 4.29 | <0.01 |
| **Comp. 23** | 10 | 0.21 |  |
| **Comp. 24** | 10 | 4.40 | <0.01 |
| **Comp. 27** | 10 | 5.26 | <0.001 |
| **Comp. 28** | 10 | 11.55 | <0.001 |
| **Comp. 30** | 10 | 5.74 | <0.001 |
| **Comp. 31** | 10 | 0.06 |  |

**Supplementary Table 18.** The Pearson correlation coefficients between the defense ability of *S. rostratum* against *L. decemlineata* and *H. armigera* and its growth traits (AEL: antifeedant activities of ethanol extracts against *L. decemlineata*; GEL: growth inhibition activities of ethanol extracts against *L. decemlineata*; AEH: antifeedant activities of ethanol extracts against *H. armigera*; GEH: growth inhibition activities of ethanol extracts against *H. armigera*. **p*<0.05, ***p*<0.01 and ****p*<0.001).

| Defense | SPAD | Pmax | SLA | H | SD | CW | RB | TB | AB | RGR |
| --- | --- | --- | --- | --- | --- | --- | --- | --- | --- | --- |
| AEL | -0.635* | -0.814** | -0.660* | -0.845*** | -0.918*** | -0.892*** | -0.839*** | -0.884*** | -0.873*** | -0.829*** |
| GEL | -0.677* | -0.840*** | -0.674* | -0.834*** | -0.878*** | -0.893*** | -0.859*** | -0.872*** | -0.866*** | -0.766** |
| AEH | 0.813** | 0.815** | 0.757** | 0.846*** | 0.945*** | 0.910*** | 0.931*** | 0.940*** | 0.949*** | 0.849*** |
| GEH | 0.568 | 0.637* | 0.328 | 0.737** | 0.809** | 0.758** | 0.654* | 0.786** | 0.771** | 0.809** |

**Supplementary Table 19.** The Pearson correlation coefficients between qualitative defensive metabolites of *S. rostratum* and its growth traits (**p*<0.05, ***p*<0.01 and ****p*<0.001).

| Compounds | SPAD | Pmax | SLA | H | SD | CW | RB | TB | AB | RGR |
| --- | --- | --- | --- | --- | --- | --- | --- | --- | --- | --- |
| **Comp. 2** | 0.408 | 0.567 | 0.701* | 0.468 | 0.486 | 0.526 | 0.616* | 0.449 | 0.495 | 0.312 |
| **Comp. 3** | 0.391 | 0.608* | 0.819** | 0.588* | 0.578* | 0.602* | 0.652* | 0.515 | 0.550 | 0.377 |
| **Comp. 4** | 0.813** | 0.841*** | 0.739** | 0.728** | 0.876*** | 0.880*** | 0.885*** | 0.872*** | 0.892*** | 0.810** |
| **Comp. 5** | 0.049 | 0.649* | 0.591* | 0.372 | 0.372 | 0.488 | 0.556 | 0.404 | 0.424 | 0.335 |
| **Comp. 6** | 0.392 | 0.664* | 0.760** | 0.444 | 0.483 | 0.563 | 0.645* | 0.476 | 0.520 | 0.361 |
| **Comp. 7** | 0.217 | 0.682* | 0.768** | 0.433 | 0.400 | 0.517 | 0.596* | 0.406 | 0.427 | 0.257 |
| **Comp. 8** | 0.020 | 0.407 | 0.358 | 0.420 | 0.246 | 0.252 | 0.322 | 0.228 | 0.261 | 0.182 |
| **Comp. 9** | 0.217 | 0.531 | 0.488 | 0.213 | 0.267 | 0.367 | 0.444 | 0.312 | 0.340 | 0.277 |
| **Comp. 10** | 0.668* | 0.862*** | 0.786** | 0.627* | 0.787** | 0.865*** | 0.877*** | 0.801** | 0.818** | 0.705* |
| **Comp. 11** | 0.184 | 0.630* | 0.597* | 0.443 | 0.432 | 0.487 | 0.546 | 0.411 | 0.434 | 0.348 |
| **Comp. 12** | 0.370 | 0.824*** | 0.611* | 0.540 | 0.615* | 0.691* | 0.717** | 0.668* | 0.673* | 0.654* |
| **Comp. 13** | 0.532 | 0.712** | 0.669* | 0.512 | 0.507 | 0.602* | 0.727** | 0.604* | 0.637* | 0.481 |
| **Comp. 14** | 0.332 | 0.487 | 0.435 | 0.465 | 0.436 | 0.429 | 0.430 | 0.379 | 0.413 | 0.360 |
| **Comp. 15** | 0.073 | 0.520 | 0.497 | 0.528 | 0.322 | 0.401 | 0.494 | 0.396 | 0.427 | 0.302 |
| **Comp. 16** | 0.259 | -0.336 | 0.071 | -0.733 | -0.044 | -0.216 | -0.100 | -0.319 | -0.323 | -0.303 |
| **Comp. 17** | 0.263 | 0.004 | 0.149 | 0.368 | 0.131 | 0.014 | 0.115 | 0.083 | 0.112 | -0.040 |
| **Comp. 18** | -0.105 | 0.062 | 0.203 | -0.126 | -0.135 | -0.046 | 0.029 | -0.107 | -0.063 | -0.136 |

**Supplementary Table 20.** The Pearson correlation coefficients between quantitative defensive metabolites of *S. rostratum* and its growth traits (**p*<0.05, ***p*<0.01 and ****p*<0.001).

| Compounds | SPAD | Pmax | SLA | H | SD | CW | RB | TB | AB | RGR |
| --- | --- | --- | --- | --- | --- | --- | --- | --- | --- | --- |
| **Comp. 19** | -0.751** | -0.703* | -0.461 | -0.438 | -0.771** | -0.821** | -0.766** | -0.823** | -0.804** | -0.812** |
| **Comp. 20** | -0.487 | -0.528 | -0.390 | -0.313 | -0.562 | -0.618* | -0.603* | -0.607* | -0.583* | -0.557 |
| **Comp. 21** | -0.768** | -0.665* | -0.568 | -0.574 | -0.814** | -0.806** | -0.775** | -0.789** | -0.785** | -0.705* |
| **Comp. 23** | -0.177 | 0.071 | 0.333 | -0.141 | -0.200 | -0.103 | 0.049 | -0.145 | -0.105 | -0.208 |
| **Comp. 24** | -0.681* | -0.836*** | -0.758** | -0.544 | -0.792** | -0.816** | -0.789** | -0.751** | -0.740** | -0.693* |
| **Comp. 26** | 0.366 | 0.346 | 0.447 | 0.173 | -0.380 | -0.742 | 0.664 | -0.184 | 0.135 | -0.168 |
| **Comp. 27** | -0.648* | -0.747** | -0.594* | -0.806** | -0.927*** | -0.866*** | -0.809** | -0.882*** | -0.864*** | -0.845*** |
| **Comp. 28** | -0.725** | -0.893*** | -0.855*** | -0.845*** | -0.953*** | -0.953*** | -0.940*** | -0.906*** | -0.918*** | -0.803** |
| **Comp. 30** | -0.593* | -0.931*** | -0.761** | -0.566 | -0.803** | -0.878*** | -0.881*** | -0.816** | -0.805** | -0.739** |
| **Comp. 31** | -0.043 | -0.154 | -0.019 | 0.057 | 0.068 | -0.007 | -0.086 | -0.019 | 0.006 | 0.077 |
